# 14 years of rotavirus A surveillance: unusual dominance of equine-like G3P[8] genotype with DS-1-like genotype constellation after the pandemic, Belgium, 2009 to 2023

**DOI:** 10.2807/1560-7917.ES.2025.30.12.2400442

**Published:** 2025-03-27

**Authors:** Mustafa Karataş, Mandy Bloemen, Lize Cuypers, Elke Wollants, Marc Van Ranst, Jelle Matthijnssens

**Affiliations:** 1 KU Leuven, Department of Microbiology, Immunology and Transplantation, Rega Institute, Laboratory of Clinical and Epidemiological Virology, Leuven, Belgium; 2 University Hospitals of Leuven, Department of Laboratory Medicine, National Reference Centre for Rotavirus, Leuven, Belgium; 3 KU Leuven, Department of Microbiology, Immunology and Transplantation, Laboratory of Clinical Microbiology, Leuven, Belgium

**Keywords:** rotavirus epidemiology, equine-like G3P[8], post-pandemic, genotype constellation

## Abstract

**Introduction:**

Despite vaccine availability, rotavirus persists as a leading cause of gastroenteritis in children younger than 5 years.

**Aim:**

We aimed to evaluate temporal changes in rotavirus epidemiology in Belgium between 2009 and 2023, including the period of the COVID-19 pandemic.

**Methods:**

We collected 8,024 rotavirus-positive stool samples throughout Belgium. For 6,352 samples, we determined the G and/or P genotypes through sequencing of the genes encoding the outer capsid proteins VP7 and VP4.

**Results:**

Before the COVID-19 pandemic, we received on average 622 samples per rotavirus epidemiological year, which decreased to 114 and 111 samples during the two pandemic rotavirus epidemiological years, followed by a peak of 1,048 samples in the first post-pandemic year. Notably, the proportion of cases in the age group 2–5-years increased from 20.3% before to 33% after the pandemic (p < 0.001). Over the 14-year study period, the most common genotypes were G2P[4], G3P[8] and G9P[8]. Post-pandemic data show an unusually strong dominance of the equine-like G3P[8] genotype which carried a DS-1-like genotype constellation in the period 2021 to 2023. Additionally, vaccinated individuals were significantly overrepresented among patients infected with the equine-like VP7 carrying G3P[8] rotavirus compared with other genotypes, including typical human VP7 G3P[8].

**Conclusion:**

Despite the presence of typical yearly genotype fluctuations, several epidemiological changes were associated with the COVID-19 pandemic, including the unusual dominance of an emerging rotavirus strain against which current vaccines may be less effective. It is essential to closely monitor this strain to determine if the phenomenon is temporary.

**Key public health message**

**What did you want to address in this study and why?**

The COVID-19 pandemic and the lockdown measures affected the epidemiology and spread of various infectious diseases worldwide. We aimed to understand how the pandemic influenced rotavirus infections in Belgium and to identify any changes in which types of rotavirus have been circulating since the pandemic. To answer this question, we used data collected 10 years before, 2 years during, and 2 years after the pandemic.

**What have we learnt from this study?**

During the pandemic, there was a substantial drop in rotavirus circulation and reported cases. However, cases strongly increased once the restrictions were lifted, especially among children aged 2–5 years. In addition, an unusual rotavirus G3-variant became very dominant in the two years after the pandemic. The vaccine currently used in Belgium might not perform as well against this new G3 variant as against other variants.

**What are the implications of your findings for public health?**

Continuous monitoring of rotavirus variants is crucial to detect new emerging variants or changes in rotavirus epidemiology with respect to successful vaccine prevention strategies. Our findings can also help understand the effects of pandemic measures on the circulation of other infectious diseases, and especially what can happen when the measures are relaxed and pathogens can start to circulate freely again.

## Introduction

Rotavirus infections constitute a considerable economic and clinical burden globally, particularly in children under 5 years of age. It was estimated that in 2019, rotavirus caused more than 1.7 million hospitalisations among children younger than 5 years with around 200,000 deaths, despite an estimated 500,000 hospitalisations prevented thanks to vaccination [1-3].

Human rotaviruses, belonging to the family *Sedoreoviridae*, genus *Rotavirus* and species *Rotavirus alphagastroenteritidis* (formerly known as rotavirus A), are characterised by a segmented double-stranded RNA genome, enclosed within three concentric capsid layers. The genotypic classification of rotaviruses primarily relies on the outer capsid proteins VP4 and VP7, which define the P and G genotypes, respectively. These proteins are pivotal in eliciting neutralising antibodies [4]. Common rotavirus genotypes G1P[8], G2P[4], G3P[8], G4P[8] and G9P[8] have been identified globally, G1P[8] being the most prevalent [5,6]. Taking these into account, Rotarix (GlaxoSmithKline), containing an attenuated human G1P[8], and RotaTeq (Merck), composed of a combination of five human-bovine reassortant strains possessing the typical human rotavirus genotypes G1, G2, G3, G4 and P[8], in combination with the bovine G6 and P[5] genotype, were developed and have been in use since 2006 [7,8]. In Belgium, the introduction of the Rotarix vaccine (administered at 2 and 3 months of age) in 2006 and RotaTeq vaccine (administered at 2, 3 and 4 months of age) in 2007 marked a milestone in rotavirus prevention, with Rotarix the predominantly used vaccine quickly achieving an uptake of over 85% [5].

The segmented nature of the rotavirus genome allows for reassortment, contributing to high genetic diversity, which may lead to the emergence of novel genotypes in the human population that may challenge the effectiveness of available vaccines. In addition, distinct genotypes co-circulate with yearly fluctuations, irrespective of whether vaccination programmes—using either Rotarix or Rotateq—are in place. [9]. However, after the implementation of Rotarix, a shift from the dominant G1P[8] genotype towards the heterotypic G2P[4] was reported in several countries [5,10-14]. When the complete rotavirus genome (11 segments) is considered, most human rotaviruses possess one of two gene constellations, often referred to as either a Wa-like or a DS-1-like genotype constellation [15]. Generally, the strains G1P[8], G3P[8], G4P[8], G9P[8] and G12P[8] are associated with the Wa-like genotype constellation, whereas the G2P[4] and G9P[4] strains are most often linked with the DS-like genotype constellation [15-20]. However, novel equine-like G3P[8] rotavirus strains have been detected in recent years, which also carry this DS-1-like genotype constellation [21,22]. Despite fluctuations in genotypes, vaccines continue to be effective in preventing severe disease and hospitalisations [23], although the overall efficacy of the monovalent rotavirus vaccine is slightly lower towards fully heterotypic genotypes, including DS-1-like G2P[4] strains [24].

During the COVID-19 pandemic, many countries, including Belgium, implemented a range of public health measures starting in March 2020. The measures included mask wearing mandates, school closures or occupancy limits, and comprehensive lockdowns, which presumably impacted transmission of most communicable diseases. Strong decreases in rotavirus infections have been reported, in some countries up to 83% fewer than in previous years [25]. However, as restrictions eased, a resurgence of rotavirus and other communicable diseases was observed [26]. This resurgence was most likely due to a combination of lifted measures resulting in strongly increased interpersonal contacts and to the lack of immune stimulation after to the reduced circulation of microbial agents during the pandemic, sometimes also referred to as immunity debt [27]. While changes in rotavirus epidemiology after the COVID-19 pandemic remain unclear, continued surveillance including genotype and vaccination information is needed to better understand the implications of the current and future outbreaks on rotavirus circulation and vaccine effectiveness.

The COVID-19 pandemic provided us with a unique natural experiment, allowing us to better understand the epidemiology of infectious diseases including rotavirus. In this study, we leveraged data from the National Reference Center (NRC) for rotavirus in Belgium, which has been running for 14 years and provides a comprehensive overview of rotavirus infections in Belgium. We aimed to reveal shifts in rotavirus epidemiology before, during and after the pandemic by analysing retrospective data from the NRC in depth.

## Methods

### Sample and data collection

Samples were collected within the framework of the NRC network for rotavirus A. Within the NRC, every hospital or general practitioner in Belgium uses their own established methods (i.e. rapid antigen test, ELISA, qPCR panel test) to determine rotavirus positivity of stool samples. Clinical hospital laboratories and diagnostic centres can voluntarily send their rotavirus-positive stool samples to the university hospital of Leuven (UZ/KU Leuven) for genotype characterisation for national surveillance purposes. For every shipped sample, related minimal patient data (e.g. date of birth, vaccination status, post code, date of sample collection) were provided to the NRC. All samples received by the NRC were subjected to genotyping. Oxford stringency index and visualisation of the data was derived from online sources [28,29].

### Shapefiles of Belgium

The shapefile of Belgium and its administrative units (version: 01.01.2023) were retrieved from the FPS Finance - General Administration of Patrimonial Documentation, Geoportal of Belgian Federal Institutions [30]. We used the shapefile to plot the distribution of received samples from each municipality in Belgium.

### Laboratory methods

#### Rotavirus genotyping of VP7 and VP4

Stool samples were diluted to a 10% concentration in phosphate-buffered saline. Viral RNA extraction was performed using the QIAamp Viral RNA mini kit (Qiagen/Westburg). The extracted RNA was denatured at 95 °C for 2 min. Reverse transcriptase PCR (RT-PCR) was conducted using the Qiagen OneStep RT-PCR Kit (Qiagen/Westburg) with previously described primers Beg9 and End9 (VP7), and VP4 1–17F and Con2Deg (VP4) [31]. We performed RT-PCR, PCR product identification, purification and sequencing as previously described [5]. Chromatogram sequencing files were quality controlled and analysed using Chromas 2.5 (Technelysium). Samples were genotyped using the National Center for Biotechnology Information (NCBI) Basic Local Alignment Search Tool (BLAST) server and the GenBank database.

#### Equine-like VP7 determination of G3P[8] sequences

All generated VP7 sequences of G3P[8] strains above 500 bp were retrieved from the NRC database. Subsequently, we performed a BLASTn search of all G3P[8] VP7 sequences against predefined reference sequences representative of typical human and equine-like G3 rotaviruses (GenBank sequences Equine-like VP7: MW280957.1, typical human VP7: MF469162.1), and used the closest hit to distinguish both G3 variants.

#### Confirmation of genotype constellations of G3P[8] and G9P[4] using next generation sequencing

A selection of 40 stool samples containing G9P[4], equine-like or typical G3P[8] strain were subjected to the NetoVIR protocol, to purify virus particles (as described previously [32]), before deep sequencing using the AVITI sequencer (Element Biosciences), with an average of 10 million 150 bp long reads per sample. Reads were quality-trimmed, assembled and taxonomically identified using the ViPER pipeline [33]. Our analyses resulted in the reliable reconstruction of the near-complete rotavirus genomes in 35 samples. Sequences were aligned with MAFFT v7 and ML tree constructed using IqTree 2.3.6 and edited on Geneious Prime (v2025.0.3) and Adobe Illustrator (2024).

### Statistical analyses

For the overall statistical analyses, we included all samples sent to the NRC as rotavirus-positive, also those for which we could not confirm rotavirus using our genotyping PCR assays. The rotavirus epidemiological year was defined as spanning from 1 August to 31 July. Depending on the specific analyses, we excluded samples for which the relevant metadata were missing from both the descriptive figures and the statistical analysis. For age group statistics and analyses on yearly distribution, this meant omitting samples with unknown dates of birth or collection, respectively. For spatial distribution figures, samples without a postcode were excluded, and for genotype figures and statistics, samples lacking genotyping results were not considered. To compile the vaccination data, individuals who received at least one dose of the vaccine were categorised as vaccinated.

To analyse differences between age groups across rotavirus epidemiological years and vaccination status compared with genotypes, we performed a z-test for proportions. Specific outlier rotavirus epidemiological years that were compared for each age group (2016/17 and 2017/18 for the ≥ 60 age group, 2021/22 for the 2–5-year age group, respectively) were not included in the expected count estimations for the test of age groups. We adjusted the p values using the Bonferroni correction method, with p < 0.01 deemed statistically significant. All analyses were conducted using RStudio (version 4.3.0) and all codes to perform the analyses and create figures are available at https://github.com/Matthijnssenslab/NRC_Belgium24.

## Results

**Figure 1:**
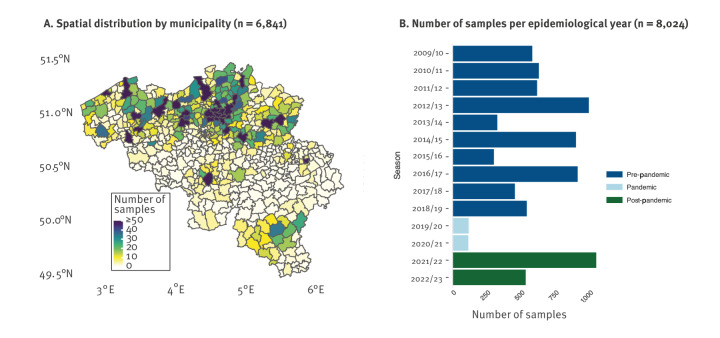
Rotavirus-positive samples, the National Reference Center, Belgium, 2009/10–2022/23

We collected 8,024 samples over the course of 14 years, between July 2009 and August 2023. The NRC collected 5,844 samples (72.8%) from Flanders (northern Belgium, ca 58% of the population), 877 samples (10.9%) from Wallonia (southern Belgium, ca 31% of the population) and 120 samples (1.5%) from the Brussels Capital Region, with 1,183 (14.7%) samples lacking postal code information (Figure 1A). Before the pandemic, a mean of 622 samples (range: 298–991) were received per rotavirus epidemiological year, whereas this number was only 114 and 111 respectively during the two pandemic rotavirus epidemiological years (2019/20 and 2020/21). However, in the first rotavirus epidemiological years after the pandemic (2021/22), a record of 1,045 samples were received (Figure 1B), whereas during the 2022/23 epidemiological year, the situation returned to pre-pandemic levels with 530 samples. Supplementary Figure S1 additionally provides the geographical distribution of samples by year. Vaccination status information was available for 63.7% (5,111) of the 8,024 samples analysed. Among these, 64.6% (3,300/5,111) were from patients vaccinated with Rotarix, 6.3% (322/5,111) were from individuals vaccinated with RotaTeq, and 29.2% (1,493/5,111) were from unvaccinated individuals. Four infants were reported as vaccinated with both RotaTeq and Rotarix and were counted in both the RotaTeq and Rotarix vaccination numbers.

### Rotavirus seasonality was disturbed during the pandemic, followed by a strong rebound

**Figure 2:**
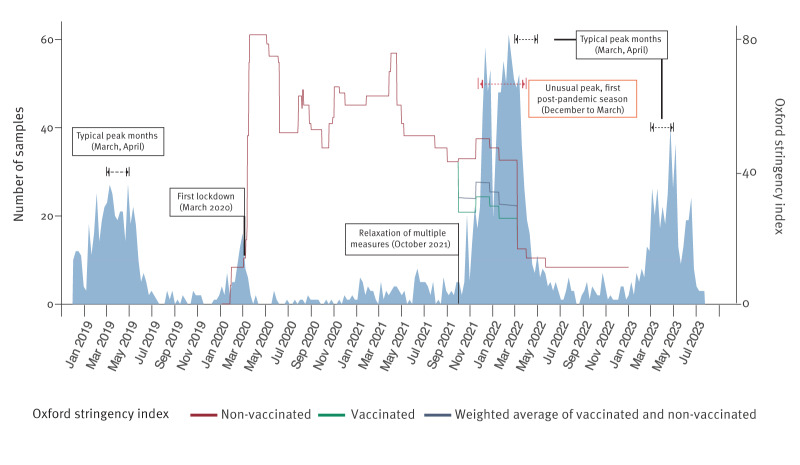
Weekly positive rotavirus samples received by the National Reference Center, Belgium, December 2018–July 2023 (n = 2,215)

Before the COVID-19 pandemic, the epidemic peak of each rotavirus season occurred in March or April, representing 20–40% of the cases per year. For a detailed breakdown of proportions of samples received per month for each epidemiological year, we refer to the appended Supplementary Figure S2. However, during the pandemic, this seasonal pattern was absent, and the number of received rotavirus positive samples was inversely correlated with the stringency of the lockdown measures, as indicated by the Oxford Stringency Index (Figure 2). In October 2021, many pandemic measures were lifted at the same time in Belgium, and schools restarted with full occupancy. Subsequently, an early and elongated peak of rotavirus cases was observed starting in December 2021. Interestingly, the peak of the second rotavirus epidemiological year after the pandemic (2022/23) occurred again between March and April, similar to that of the pre-pandemic epidemiological years, although it had a small tail until June.

### High prevalence of 2–5-year-old rotavirus cases after the pandemic

**Figure 3:**
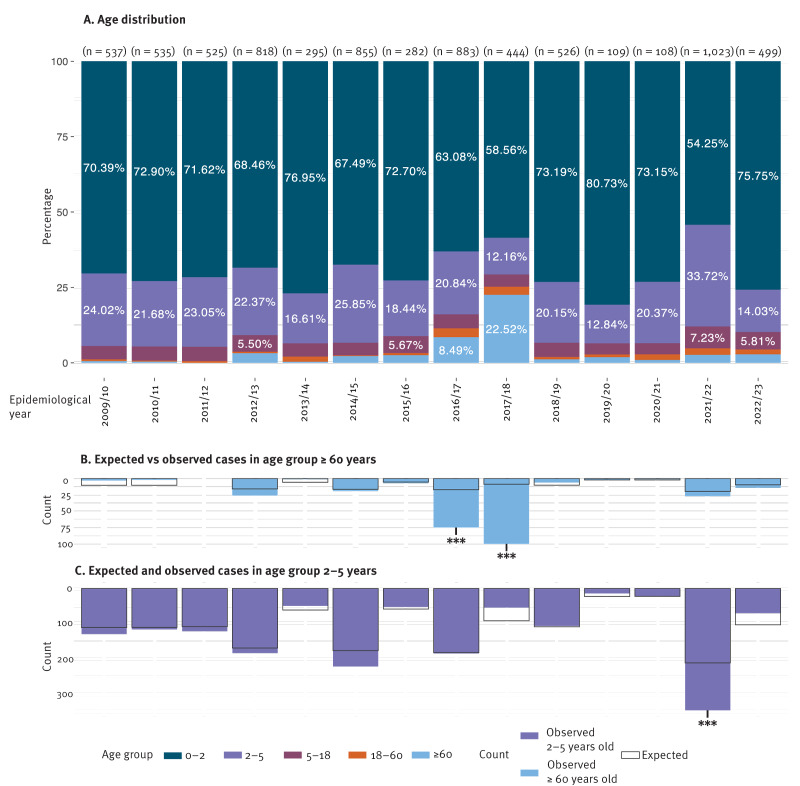
Rotavirus-positive samples by age group and rotavirus epidemiological year, Belgium, 2009/10–2022/23 (n = 7,439)

During the 14 rotavirus epidemiological years, an average of 70.1% (range: 54.3–80.7%) of the cases were infants between the ages of 0 and 2 years (Figure 3A), followed by children aged 2–5 years with an average of 20.3% of the cases (12.0–33.2%). Only 4.8% (3.6–7.2%) and 3.7% (0.33–22.3%) of the cases belonged to the age groups 5–18-years and ≥ 60 years, respectively. For the ≥ 60-year-olds, the 2016/17 and 2017/18 epidemiological years were statistically significant outliers with 8.4% and 22.3% of the cases, respectively (Figure 3A and B). Upon further inspection, we found that almost all the samples from this age group were sent to the NRC by the same hospital and were derived from a retirement home close to Mechelen. A second observation was that during the COVID-19 pandemic (2019/20 and 2020/21), the age distribution of rotavirus cases was similar to previous years. However, in the 2021/22 epidemiological year, after the COVID-19 measures were relaxed, the group of 2–5-year-old children was significantly overrepresented compared with the average of the preceding 12 epidemiological years (p < 0.001, Figure 3C). Additional spatial analysis data for the ≥ 60 years-old age group and raw data for sample counts for all age groups per epidemiological year are provided in Supplementary Figure S3 and Supplementary Table S4C, respectively.

Tests: z-test for proportions, adjusted with Bonferroni correction (***, p < 0.001). Samples without age information (no collection date or birth date) were excluded from the figures above.

### Most common genotypes from 2009 to 2023: G3P[8], G2P[4] and G9P[8] 

**Figure 4:**
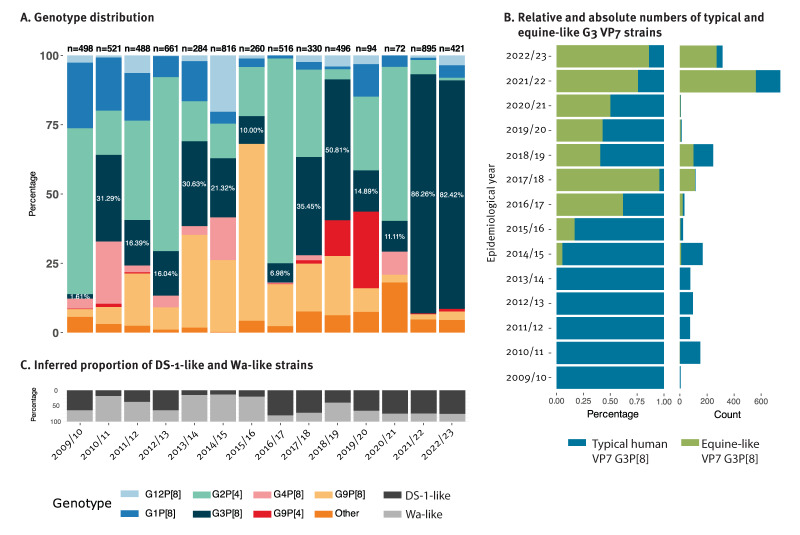
Rotavirus genotypes by rotavirus epidemiological year, Belgium, 2009/10–2022/23 (n = 6,352)

Overall, for 6,352 of 8,024 samples (79.2%), genotyping of either VP7 or VP4 was successful. During 14 years, the four most common genotypes were G3P[8] (n = 2,190; 34.5%), G2P[4] (n = 1,778; 28.0%), G9P[8] (n = 944; 14.9%) and G1P[8] (n = 496; 7.8%). While the G1P[8] genotype was still responsible for 7.5–23.6% of the cases between 2009 and 2014, its prevalence decreased below 5% in eight of the nine subsequent (2015–2023) epidemiological years (Figure 4A). Despite strong fluctuations, the G2P[4] genotype was the most common genotype between 2009 and 2021. The G9P[8] genotype prevalence increased steadily from 2009, reaching peak dominance in 2015/16 (63.8%), after which it gradually decreased again, although a notable temporary emergence of the G9P[4] genotype occurred between 2018 and 2020. For a detailed breakdown of genotypes per rotavirus epidemiological year we refer to the appended Supplementary Table S4A.

For ‘Other’ genotypes no assumption of the genotype constellations were attempted, and therefore they were excluded from Panel C.

### Dominance of equine-like G3 strains in Belgium after the COVID-19 pandemic

Between 2009 and 2021, the G3P[8] genotype showed strong fluctuations, reaching a prevalence of over 50% in the 2018/19 rotavirus epidemiological year. However, it completely dominated the two post-pandemic rotavirus epidemiological years with 86.3% and 82.4% prevalence (Figure 4.A). Such dominance, and especially in two consecutive years, had not been observed before in Belgium. Given that in recent years, the G3P[8] genotype is known to circulate with either a Wa-like genotype constellation (typical human G3) or a DS-1-like genotype constellation (often referred to as equine-like G3), we further analysed the VP7 sequences of all our G3P[8] strains, revealing a steady increase in the dominance of this equine-like G3 genotype over the typical G3 strain as of 2014 (Figure 4.B). In absolute numbers, these equine-like G3P[8] strains caused 561 and 271 cases in the 2021/22 and 2022/23 epidemiological years, respectively. For the 2021/22 epidemiological year, this was the highest number of cases reported for a single genotype during our entire study period. For detailed genotype information including G3, equine-like G3 and other genotypes we refer to Supplementary Tables S4A, S4B and S4D.

### Increasing dominance of DS-1-like rotavirus strain over Wa-like strains

**Figure 5:**
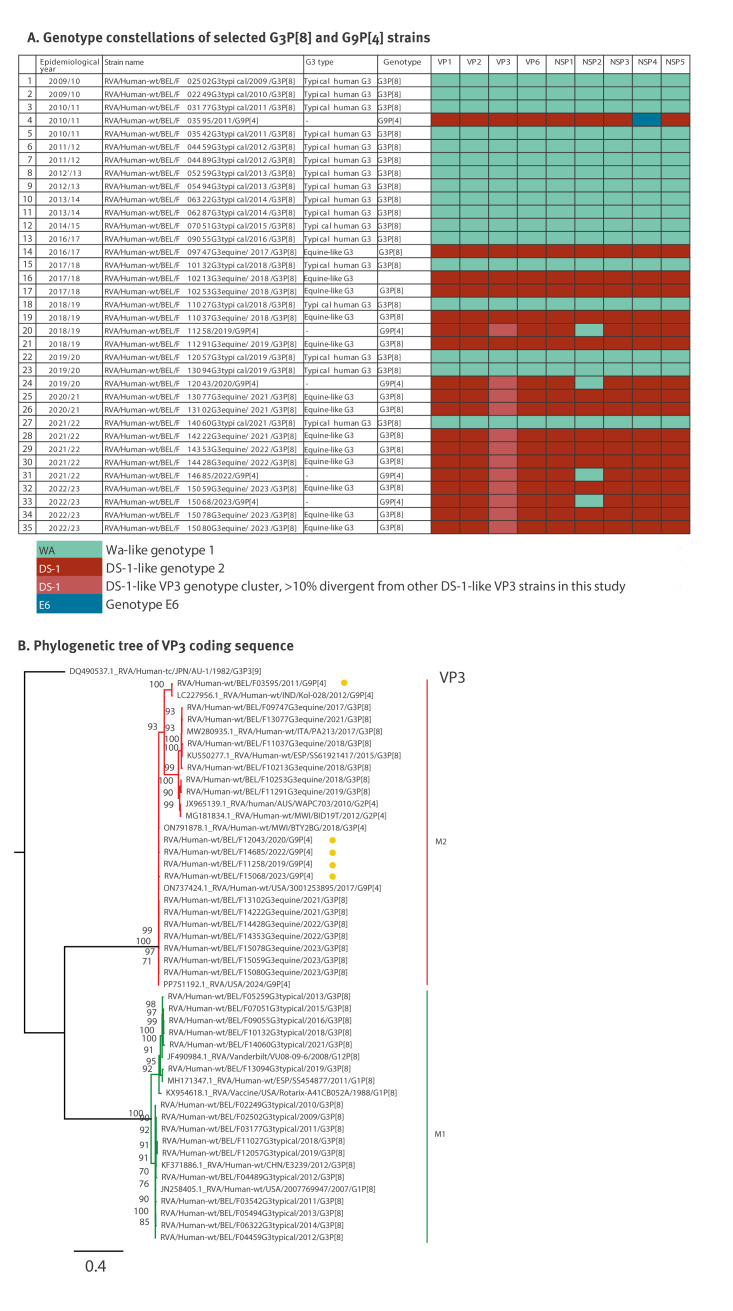
Rotavirus genotype constellations by rotavirus epidemiological year and VP3 phylogeny, Belgium, 2009/10–2022/23 (n = 35)

The predominance of G3P[8] strains with a presumed DS-1-like genotype constellation prompted a literature review of the most likely genotype constellation associated with various rotavirus G/P-genotype combinations. This search indicated that G1P[8], typical human G3P[8], G4P[8], G9P[8] and G12P[8] strains are usually found with a Wa-like genotype constellation, whereas G2P[4], the equine-like G3P[8] and G9P[4] strains are usually found with a DS-1-like genotype constellation [15-17,21,22]. When coupling this information with genotype prevalence data from our 14-year study period, we observed that the Wa-like strains dominated five of the first seven years (2009–2016) of our surveillance, whereas DS-1-like strains dominated six of the seven epidemiological years (2016–2023) at the end of our study period (Figure 4C). To further confirm our genotype constellation assumption with respect to genotypes G9P[4] and G3P[8], we determined the near complete genomes of rotavirus from 35 samples throughout the study period. As expected, sequencing results showed that all 13 equine-like G3P[8] strains carried the DS-1-like genotype constellations, whereas all 17 typical human G3P[8] strains exhibited the Wa-like genotype constellations (Figure 5A). On the other hand, the G9P[4] sample from 2011 possessed a reassortant NSP4 gene (typed as E6) in a DS-1-like genotype constellations, whereas the four more recently identified G9P[4] strains showed a reassortant Wa-like NSP2, inside a DS-1-like genotype constellation (Figure 5A). Further phylogenetic analyses revealed that the VP3 gene segment of the post-pandemic equine-like G3P[8] clustered with G9P[4] strains detected after 2019 and showed 10% divergence compared with pre-pandemic equine-like G3P[8] VP3 (Figure 5B). Phylogenetic trees of all segments can be found in Supplementary Figures S5.1–11.

Panel B: Alignment done with MAFFT v7; ML tree constructed using IqTree (v2.3.6) with 1,000 bootstraps. Red: DS-1-like segment. Green: Wa-like segment. Yellow dots: G9P[4]. Strain names including an accession number were retrieved from GenBank.

### Significantly higher proportion of equine-like G3P[8] in vaccinated infants

**Figure 6:**
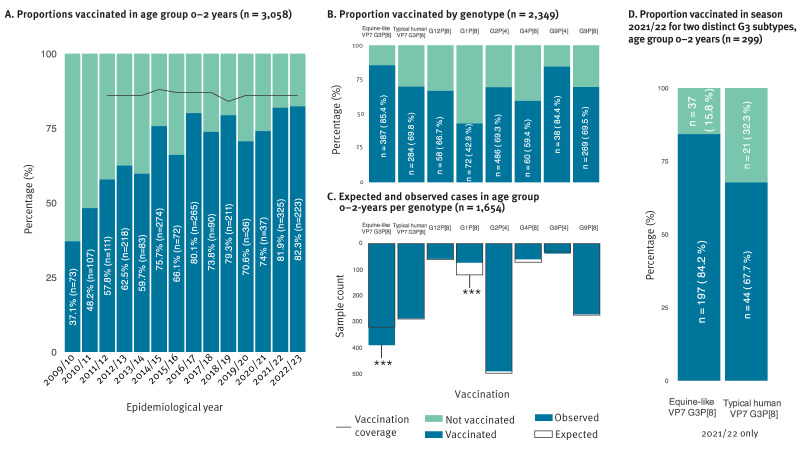
Proportions of vaccinated infants (0–2 years) over the years and for different genotypes 2009/10–2022/23

Over the course of the 14 years of rotavirus NRC activities, we observed a steady increase in the proportion of 0–2-year-old infants who received at least one Rotarix vaccine dose, while the overall estimated vaccination coverage was stable between 2011 and 2022 (Figure 6A). We compared proportions of vaccinated and unvaccinated infants per genotype, (splitting typical human and equine-like G3 strains) (Figure 6B) and compared this to what would be expected if the vaccinated group proportion was the same in each genotype, which should be the case if the vaccine protects against all genotypes equally well. Not unexpectedly, the vaccinated group was under-represented in infections with the G1P[8] genotype (p < 0.001, expected: 118, observed: 72), as this genotype is homotypic with the Rotarix vaccine strain (Figure 6C). Surprisingly, the opposite was observed for the group infected with the equine-like G3 strain, where vaccinated infants were over-represented (p < 0.001, expected: 318, observed: 387) (Figure 6C). To check if this finding could be an artefact of the COVID-19 pandemic and/or the dominance of G3P[8] after the pandemic, we performed the same analysis only for the epidemiological year 2021/22 and observed the same phenomenon: samples from vaccinated infants encompassed 84.2% (197/234) of the equine-like VP7 G3P[8] and 67.7% (44/65) of the typical human VP7 G3P[8] genotyped samples (Figure 6D). No difference between equine-like and typical G3P[8] strain distributions were observed per age groups. Counts of typical and equine-like G3P[8] genotype in different age groups per rotavirus epidemiological year can be found in Supplementary Figure S6.

Panel A: Vaccination coverage was retrieved from World Health Organization data [48], immunisation coverage estimates by country (2022). Panel C: We assumed that the vaccines protected equally well against all genotypes. Test: Z-test for proportions, adjusted using Bonferroni correction (**, p < 0.001).

## Discussion

Global studies have highlighted a marked decline in the circulation of communicable pathogens such as RSV, influenza virus, *Streptococcus pneumoniae* and others during the COVID-19 pandemic, only to face a rebound later [34-36]. Our research echoes these findings, showing a significant drop in rotavirus-positive samples sent for genotyping during the pandemic, with a surge post-pandemic, the highest since our NRC was initiated. Under normal circumstances, rotaviruses are believed to be ubiquitous in the environment and the human population, and infants are believed to be frequently re-exposed to wild-type rotavirus infections (or vaccination), repeatedly boosting their immunity [37]. We suggest that relaxed measures and lack of immune boosting among children during the lockdown period led to an increase in rotavirus spread and subsequent clinical manifestations once the pandemic restrictions were lifted.

Historically, the rotavirus peak incidence in Belgium fell between December and February before vaccine introduction. After the vaccine was introduced in 2006, this peak shifted towards March and April [5,38]. Our data from 2009 to 2019 support this post-vaccination seasonal trend. However, pandemic measures (quantified using the Oxford stringency index) nearly flatlined this pattern between March 2020 until October 2021 (Figure 2), followed by a strong resurgence right after relaxation of measures. The following rotavirus season began earlier than usual (December), most likely due to the accumulation of susceptible infants, resulting in the observed strong and long rotavirus season. By the 2022/23 rotavirus epidemiological year, the seasonal pattern seemed to return to normal albeit it slightly prolonged to June.

The age distribution of rotavirus A cases around the world is rather consistent, affecting mostly children younger than 5 years, followed by the population ≥ 60 years. Interestingly, the latter group contributed unusually many rotavirus-positive samples in the rotavirus epidemiological years 2016/17 and 2017/18. Further analyses showed that these samples were derived from patients who were registered as resident in various neighbouring municipalities, but who in practice lived together in the same retirement home. All these samples were sent by the same doctor and after consulting them, these turned out to be outbreaks in a retirement home, which was detected because of a temporary change in the testing strategy (from a norovirus-specific test to a combined norovirus/rotavirus test). In general, rotavirus testing is not routinely included in gastroenteritis outbreaks in the ≥ 60-year-old population in Belgium, as only testing for the 0–5-year age group is reimbursed. This case suggests that rotavirus outbreaks in this population might remain largely undiagnosed. On the other hand, during the first year after the pandemic, there was a notable increase in the predominance in the samples from children aged 2–5 years, suggesting that the reduced exposure to rotavirus during the pandemic impacted rotavirus circulation in this age group more than in the 0–2-year-old group.

Rotarix was introduced in Belgium in 2006, and vaccination rates were close to 90% in subsequent years, resulting in substantial changes in the epidemiology of rotavirus in Belgium [5]. Before the vaccine introduction, the homotypic G1P[8] was the predominant genotype, worldwide as well as in Belgium, whereas after the vaccination, a shift towards the G2P[4] genotype was observed, not just in Belgium but also in other countries where Rotarix is the main vaccine used [5,10-14].

Rotavirus vaccination has been estimated to reduce rotavirus fatalities from 528,000 in 2000 to 215,000 in 2013, highlighting the significant impact of extensive rotavirus vaccination [39]. While genotype proportions tend to fluctuate over time and geographic regions, the monovalent rotavirus vaccine Rotarix continues to be effective in preventing severe disease and hospitalisations, despite its slightly lower effectiveness against G2P[4] [24], which might explain the fluctuating but high prevalence of G2P[4] in Belgium post vaccine introduction. Interestingly, we observed a gradual increase in the number of vaccinated patients’ samples sent to the NRC, while vaccination coverage was stable in the same age group during the study period, coinciding with the increase in the number of equine-like G3P[8] strains. Our in-depth analysis shows that the sample proportion of vaccinated patients was higher in equine-like VP7-containing G3P[8] strains, which suggests that Rotarix might be slightly less effective against equine-like G3P[8] strains than other genotypes, which might (partially) explain the high G3P[8] dominance in Belgium after the pandemic.

This equine-like VP7 G3P[8] genotype has been strongly associated with the DS-1-like genotype constellation [21,22,40] and became dominant especially between 2017 and 2020 in many countries including Brazil, Italy, Botswana, Malaysia and Thailand [41-45]. Similar to other studies, our study shows this consistent predominance over the years. In Thailand, Tacharoenmuang et al. showed that the VP6 gene segment of equine-like G3P[8] clustered with the VP6 of local G2P[4] strains, suggesting local reassortment events in these DS-1-like strains [45]. Moreover, Katz et al. also noted multiple gene reassortments between locally circulating equine-like G3P[8] and G2P[4] strains in Dominican Republic [46]. In our study, we show reassortment of the VP3 gene between locally circulating equine-like G3P[8] and G9P[4] strains circulating in the same rotavirus epidemiological years, further showcasing the dynamic nature of rotavirus genomes. The presence of this reassortant was only observed in the post-pandemic period and coincided with the absolute dominance of G3P[8] genotype. While VP3 is a multifunctional protein with several activities that counter anti-rotavirus immune responses, it is not clear if the observed dominance of equine-like G3P[8] strains in the post-pandemic period can be attributed to this VP3 reassortment.

Extrapolation of the rotavirus genotype constellation based on the available G/P genotype sequences (and confirmed by sequencing of G9P[4] and G3P[8] strains), identified a strong dominance of DS-1-like strains in the last seven rotavirus epidemiological years. This finding seems to confirm a prediction we made more than 10 years ago, that a relative enrichment of DS-1-like RVA strains (replacing the Wa-like constellation present in the Rotarix vaccine), might occur over time due to vaccine pressure [47]. Recently, Degiuseppe et al. also reported that in four of the seven South American countries, there was a rapid switch from Wa-like constellation to DS-1-like constellation, despite distinct vaccine introduction trends as well as different vaccine types in each of the countries [12].

Moreover, an increase in the proportion of rotavirus-positive samples from vaccinated infants over time and a higher incidence of vaccinated infants among cases infected with equine-like G3P[8] strains, align with possible positive selection of DS-1-like strains. It could be argued that this phenomenon cannot be explained only by this DS-1-like genotype constellation, as we did not observe an over-representation of DS-1-like G2P[4] strains in the vaccinated infants. However, it could be counter-argued that the prolonged circulation of G2P[4] strains in the Belgian population after vaccine introduction might have resulted in a good population immunity against the G2 VP7, which is not (yet) present against the equine-like G3 VP7. We speculate that the current dominance of the equine-like G3 strains in Belgium is a result of vaccine pressure towards DS-1-like strains, random stochastic variations and a bottleneck event cause by the pandemic.

In our study, several limitations should be acknowledged: (i) incompleteness of clinical data (e.g. vaccination data, sex distribution), as this information is not always complete in national databases; (ii) possible geographical selection bias due to the voluntary nature of sample submission to the NRC and more samples received from Flanders (north of Belgium); (iii) possible temporal sample bias due to changes occurring in participating hospitals. However, the number of hospitals sending us rotavirus samples has been relatively stable over time, and also during and after the pandemic no notable changes have been observed. Therefore, we do believe that the samples we receive are representative for the true rotavirus epidemiology in Belgium. (iv) Although national reimbursement policies for rotavirus tests remained unchanged, testing practices may have fluctuated, as seen during outbreaks in the  ≥ 60-year-old population from 2016 to 2018. However, we did not observe any unusual activity at specific testing centres. A notable strength of our study is its status as the largest nationwide assessment of rotavirus epidemiology in the context of a pandemic.

## Conclusion

Overall, we recommend that surveillance programmes extend their focus beyond the VP7 and VP4 genotypes, towards other rotavirus gene segments. Such expansion could provide crucial insights into the impact of vaccination on the evolution of rotavirus genotypes, and contribute to the development of next-generation vaccines that consider the full range of protective antigens. Our findings highlight the evolving nature of rotavirus A, and a need for future research to explore how the virus adapts in response to changes in vaccine policies and public health emergencies.

## Supplementary Data

9083000 Supplement
