# Supplementary material for: 14 years of rotavirus A surveillance: unusual dominance of equine-like G3P[8] genotype with DS-1-like genotype constellation after the pandemic, Belgium, 2009 to 2023
Source: Euro Surveill. 2025 Mar 27;30(12):2400442. doi: 10.2807/1560-7917.ES.2025.30.12.2400442 (PMC11951416; doi:10.2807/1560-7917.ES.2025.30.12.2400442)
Supplement: Supplement [file 24-00442_MATTHIJNSSENS_Supplement.pdf]

This supplementary material is hosted by Eurosurveillance as supporting information alongside the article "14 years of rotavirus A surveillance: unusual dominance of equine-like G3P[8] genotype with DS-1-like genotype constellation after the pandemic, Belgium, 2009 to 2023", on behalf of the authors, who remain responsible for the accuracy and appropriateness of the content. The same standards for ethics, copyright, attributions and permissions as for the article apply. Supplements are not edited by Eurosurveillance and the journal is not responsible for the maintenance of any links or email addresses provided therein.

Supplementary information S1. Rotavirus samples received from every municipality in Belgium over the course of 14 years. **(n=6841)**

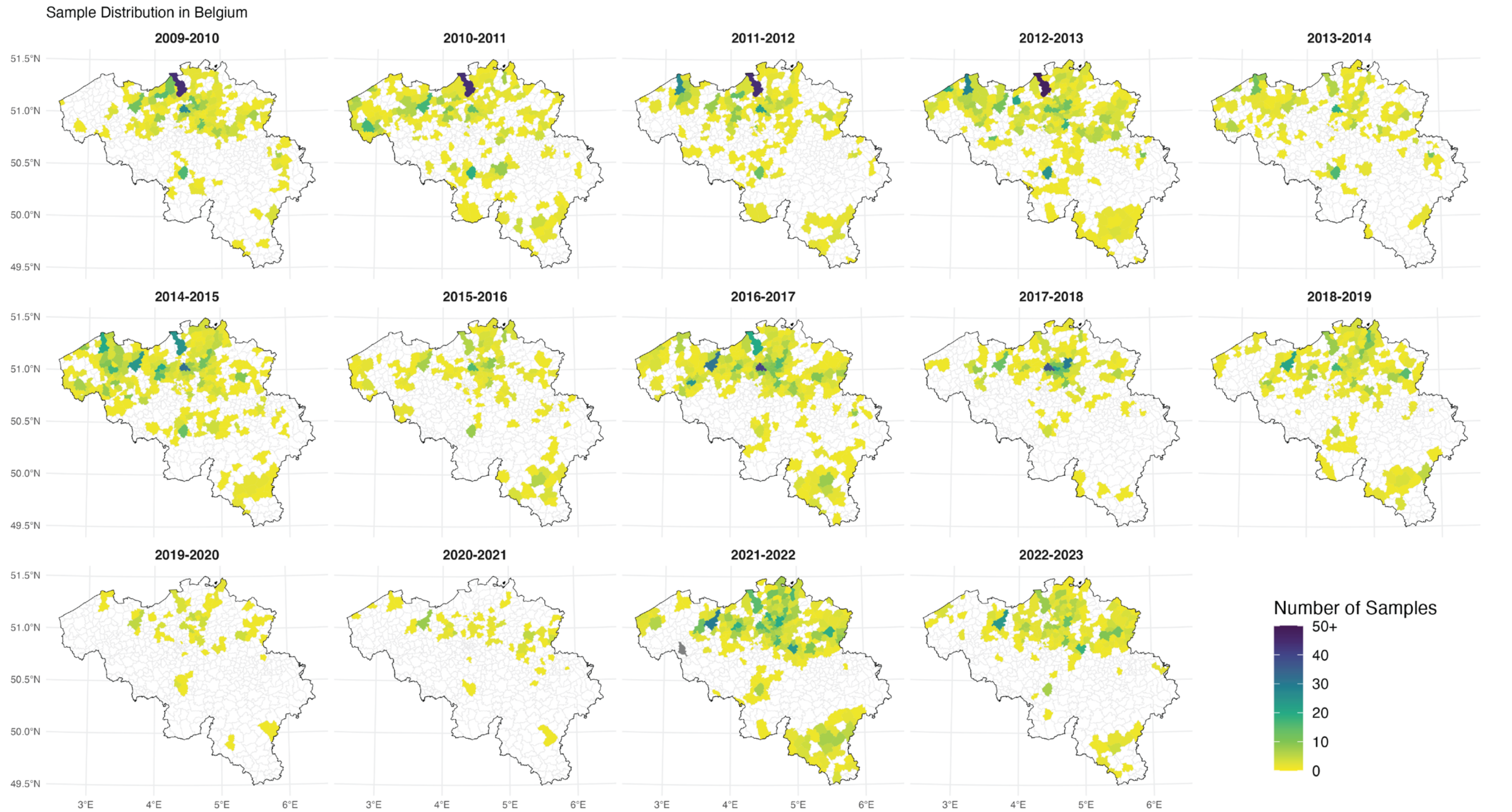

This supplementary material is hosted by Eurosurveillance as supporting information alongside the article "14 years of rotavirus A surveillance: unusual dominance of equine-like G3P[8] genotype with DS-1-like genotype constellation after the pandemic, Belgium, 2009 to 2023", on behalf of the authors, who remain responsible for the accuracy and appropriateness of the content. The same standards for ethics, copyright, attributions and permissions as for the article apply. Supplements are not edited by Eurosurveillance and the journal is not responsible for the maintenance of any links or email addresses provided therein.

Supplementary information S2. Seasonality of cases per season. Highest percentage of samples received was shown with a point.

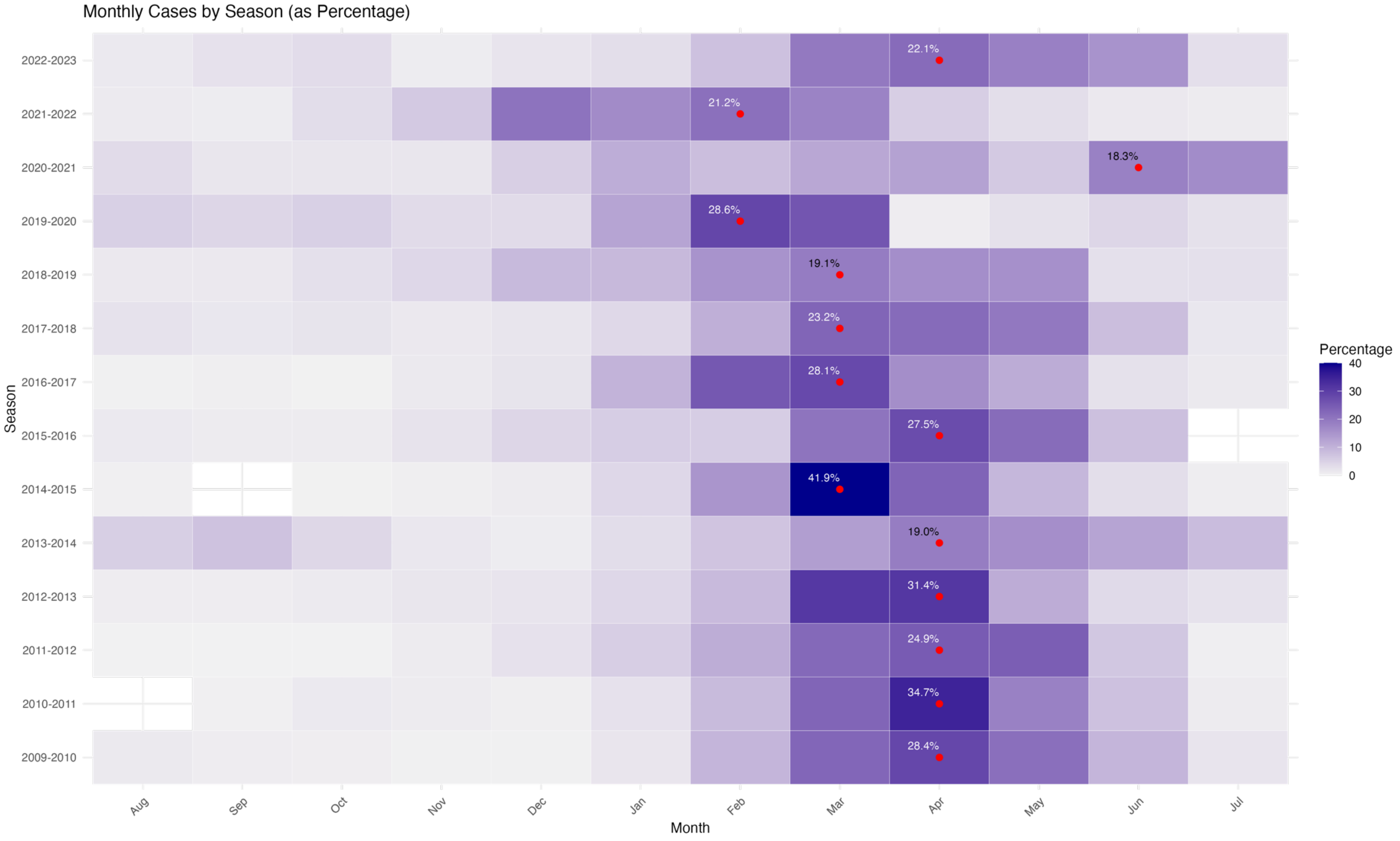

This supplementary material is hosted by Eurosurveillance as supporting information alongside the article "14 years of rotavirus A surveillance: unusual dominance of equine-like G3P[8] genotype with DS-1-like genotype constellation after the pandemic, Belgium, 2009 to 2023", on behalf of the authors, who remain responsible for the accuracy and appropriateness of the content. The same standards for ethics, copyright, attributions and permissions as for the article apply. Supplements are not edited by Eurosurveillance and the journal is not responsible for the maintenance of any links or email addresses provided therein.

Supplementary information S3. 60+ cases in 2016-2017 and 2017-2018 seasons.

2017-2018

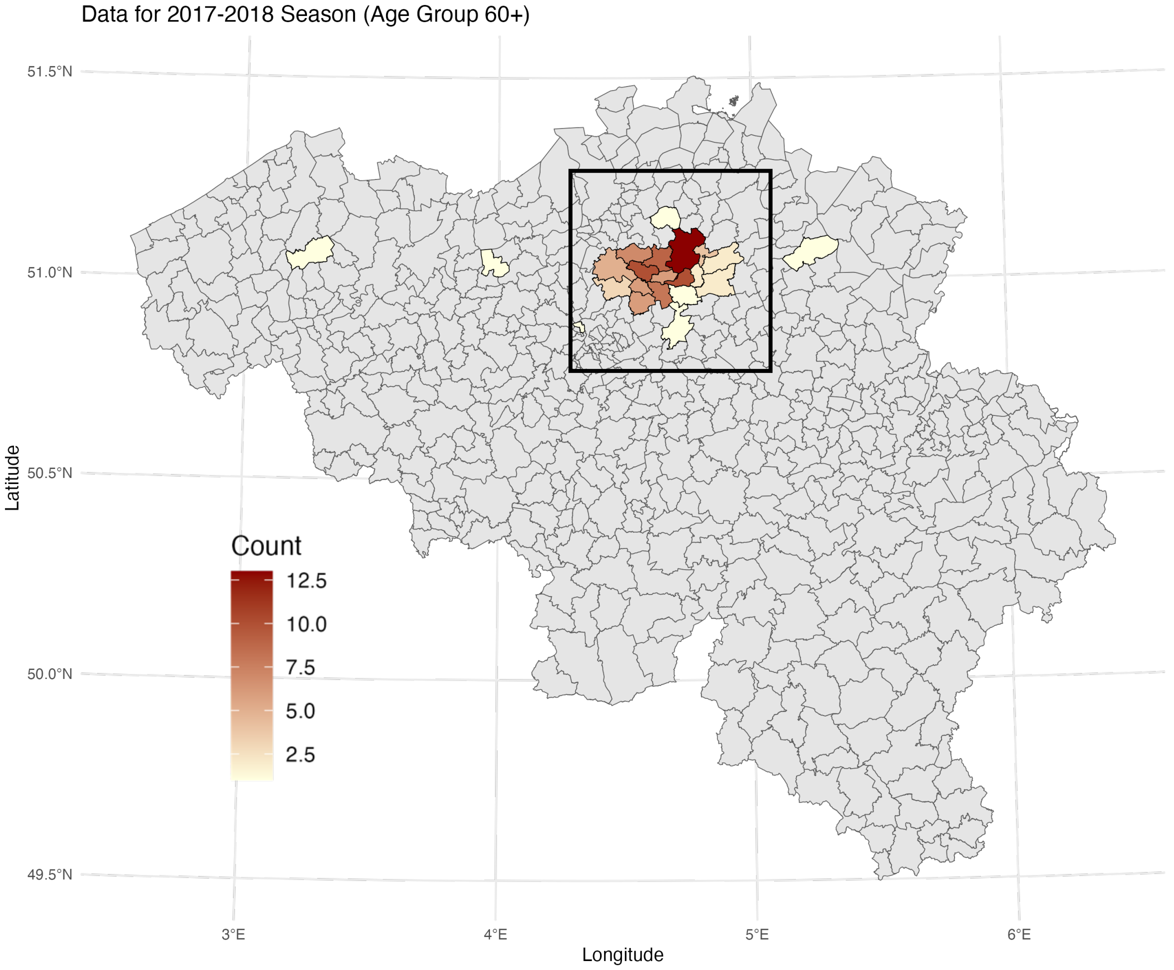

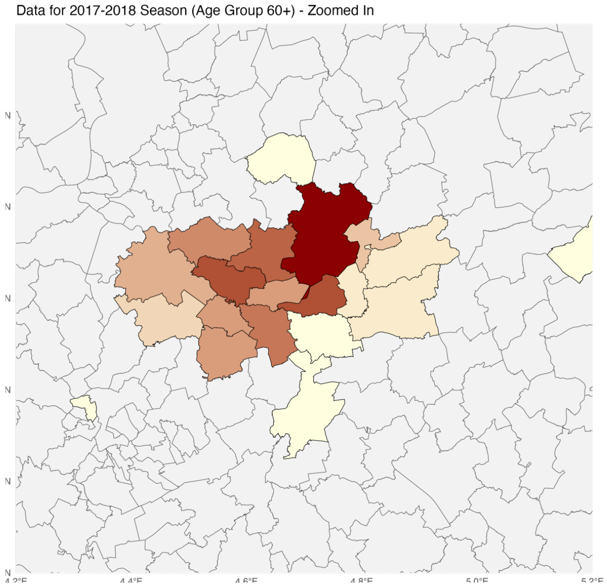

100/444 samples (25%) in 2017-2018 were received from patients older than 60.

This supplementary material is hosted by Eurosurveillance as supporting information alongside the article "14 years of rotavirus A surveillance: unusual dominance of equine-like G3P[8] genotype with DS-1-like genotype constellation after the pandemic, Belgium, 2009 to 2023", on behalf of the authors, who remain responsible for the accuracy and appropriateness of the content. The same standards for ethics, copyright, attributions and permissions as for the article apply. Supplements are not edited by Eurosurveillance and the journal is not responsible for the maintenance of any links or email addresses provided therein.

2016-2017

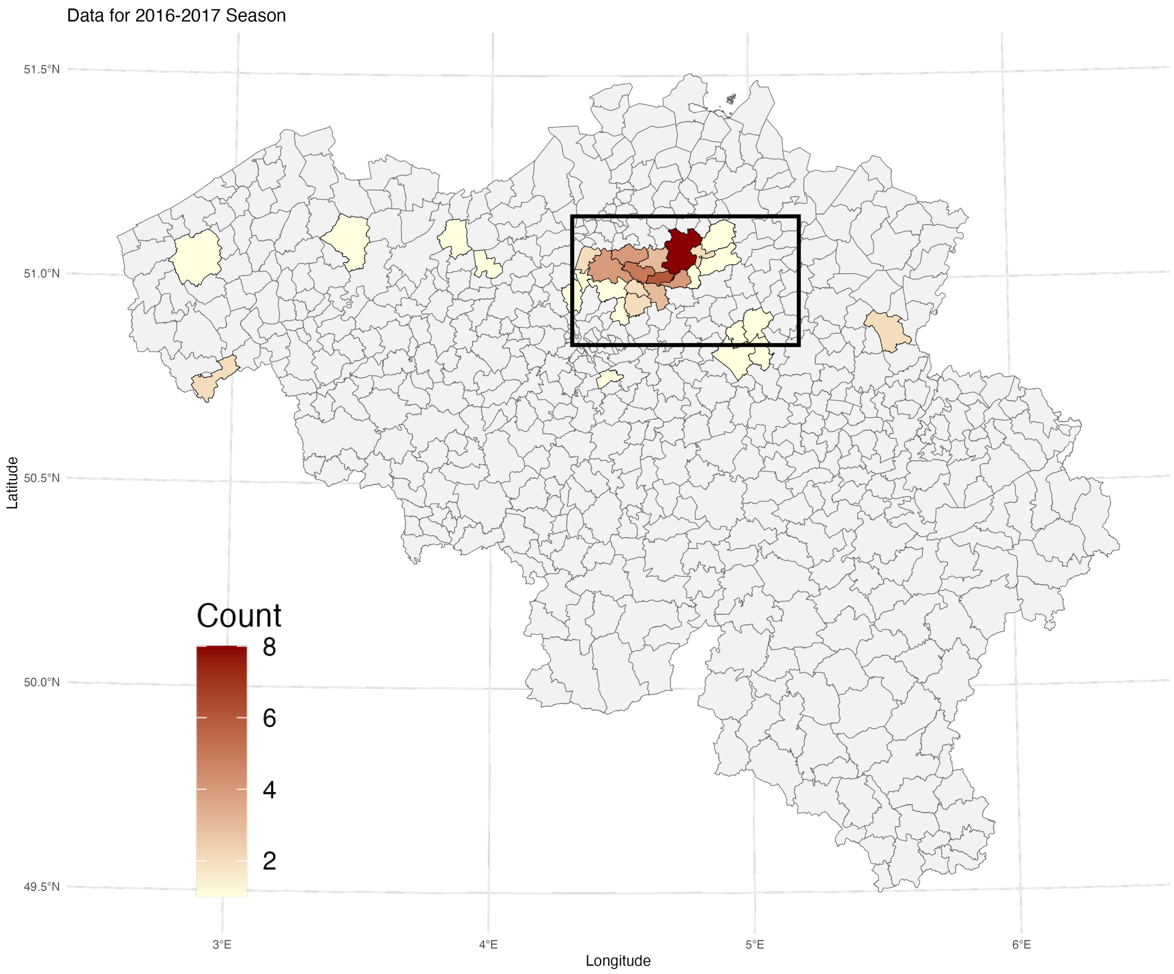

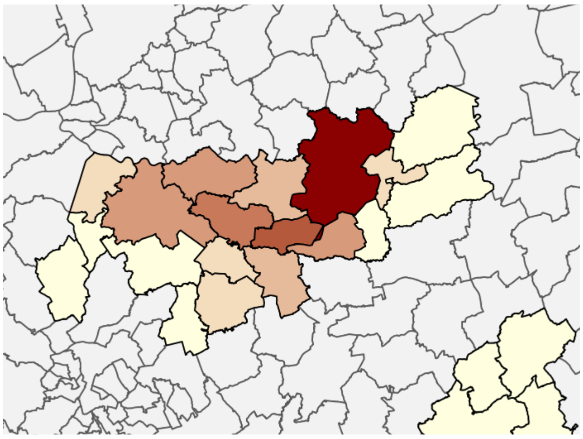

75/883 (8%) samples in 2017-2018 were received from patients older than 60.

This supplementary material is hosted by Eurosurveillance as supporting information alongside the article "14 years of rotavirus A surveillance: unusual dominance of equine-like G3P[8] genotype with DS-1-like genotype constellation after the pandemic, Belgium, 2009 to 2023", on behalf of the authors, who remain responsible for the accuracy and appropriateness of the content. The same standards for ethics, copyright, attributions and permissions as for the article apply. Supplements are not edited by Eurosurveillance and the journal is not responsible for the maintenance of any links or email addresses provided therein.

Supplementary information S4. Genotype and age group information by season.

S4.A. Genotypes

Season	G12P[8]	G1P[8]	G2P[4]	G3P[8]	G4P[8]	G9P[4]	G9P[8]	Genotyping not possible	*"Other" in figure 5*		Total
									Vaccine-derived G1P[8]	Other	
2009-2010	13	118	298	8	18	1	14	80	0	28	578
2010-2011	4	100	83	163	117	6	32	105	0	16	626
2011-2012	31	84	175	80	12	2	92	125	0	12	613
2012-2013	2	50	415	106	28	0	53	330	0	7	991
2013-2014	6	41	41	87	9	0	95	39	0	5	323
2014-2015	166	35	102	174	126	0	211	81	0	2	897
2015-2016	3	8	46	26	0	0	166	38	9	2	298
2016-2017	0	6	381	36	2	1	78	393	7	5	909
2017-2018	8	9	104	117	6	4	57	121	24	1	451
2018-2019	20	5	18	252	0	64	106	42	7	24	538
2019-2020	3	11	25	14	0	26	8	20	0	7	114
2020-2021	0	3	40	8	6	0	2	39	10	3	111
2021-2022	8	7	46	772	0	3	17	150	5	37	1045
2022-2023	15	19	4	347	0	4	13	109	0	19	530
Total	**279**	**496**	**1778**	**2190**	**324**	**111**	**944**	**1672**	**62**	**168**	**8024**

S4.B. Typical and equine like VP7 G3P[8] proportions per year

season	*Typical VP7*	*Equine-like VP7*
2009-2010	6	0
2010-2011	150	0
2011-2012	75	0
2012-2013	97	0
2013-2014	77	0
2014-2015	159	9
2015-2016	20	4
2016-2017	13	21
2017-2018	5	111
2018-2019	146	100
2019-2020	8	6
2020-2021	4	4
2021-2022	180	561
2022-2023	44	271

#Sequences shorter than 500 nucleotides were excluded from the table above.

S4.C. Age groups

season	*0-2*	*2-5*	*5-18*	*18-60*	*60*	*NA*	*Total*
2009-2010	378	129	24	3	3	41	**578**
2010-2011	390	116	25	2	2	91	**626**
2011-2012	376	121	25	3	0	88	**613**
2012-2013	560	183	45	4	26	173	**991**
2013-2014	227	49	13	5	1	28	**323**
2014-2015	577	221	36	2	19	42	**897**
2015-2016	205	52	16	2	7	16	**298**
2016-2017	557	184	41	26	75	26	**909**
2017-2018	260	54	18	12	100	7	**451**
2018-2019	385	106	25	4	6	12	**538**
2019-2020	88	14	4	1	2	5	**114**
2020-2021	79	22	4	2	1	3	**111**
2021-2022	555	345	74	22	27	22	**1045**
2022-2023	378	70	29	8	14	31	**530**

This supplementary material is hosted by Eurosurveillance as supporting information alongside the article "14 years of rotavirus A surveillance: unusual dominance of equine-like G3P[8] genotype with DS-1-like genotype constellation after the pandemic, Belgium, 2009 to 2023", on behalf of the authors, who remain responsible for the accuracy and appropriateness of the content. The same standards for ethics, copyright, attributions and permissions as for the article apply. Supplements are not edited by Eurosurveillance and the journal is not responsible for the maintenance of any links or email addresses provided therein.

S4.D. Other genotypes

season	G12P[6]	G1P[4]	G1P[x]	G2P[x]	G3P[6]	G6P[14]	G9P[6]	GxP[4]	GxP[8]	G2P[8]	G3P[4]	G4P[4]	G6P[8]	G6P[5]	G2P[6]	G3P[14]	G3P[9]	G10P[14]	G38P[28]	G4P[6]	G8P[8]	G1P[6]	P[8]Gx	GXP[4]	GXP[8]	G3P[x]	P[4]Gx	P[x]G9	G3P[3]
2009-2010	1	4	1	9	1	2	1	7	2	0	0	0	0	0	0	0	0	0	0	0	0	0	0	0	0	0	0	0	0
2010-2011	3	3	0	0	0	1	0	0	0	5	2	1	1	0	0	0	0	0	0	0	0	0	0	0	0	0	0	0	0
2011-2012	2	2	0	0	0	1	0	0	0	4	1	0	1	1	0	0	0	0	0	0	0	0	0	0	0	0	0	0	0
2012-2013	0	1	0	0	0	0	0	0	0	1	1	0	1	0	1	1	1	0	0	0	0	0	0	0	0	0	0	0	0
2013-2014	0	0	0	0	0	0	0	0	0	0	1	0	0	0	0	0	0	1	1	2	0	0	0	0	0	0	0	0	0
2014-2015	0	0	0	0	0	0	0	0	0	1	0	1	0	0	0	0	0	0	0	0	0	0	0	0	0	0	0	0	0
2015-2016	0	0	0	0	0	0	0	0	0	0	0	0	0	0	0	1	0	0	0	0	1	0	0	0	0	0	0	0	0
2016-2017	0	0	0	0	0	1	0	0	0	0	1	0	0	0	0	0	0	0	0	0	2	1	0	0	0	0	0	0	0
2017-2018	0	0	0	0	0	0	0	0	0	0	1	0	0	0	0	0	0	0	0	0	0	0	0	0	0	0	0	0	0
2018-2019	0	0	0	0	0	0	0	0	0	0	5	0	0	0	0	1	0	0	0	1	16	0	1	0	0	0	0	0	0
2019-2020	2	0	0	0	0	0	0	0	0	0	2	0	1	0	0	0	0	0	0	0	2	0	0	0	0	0	0	0	0
2020-2021	0	0	0	1	0	0	0	0	0	0	0	0	0	0	0	0	0	0	0	0	0	0	0	1	1	0	0	0	0
2021-2022	0	0	0	5	0	0	0	0	14	0	2	0	0	0	0	0	0	0	0	0	1	0	0	0	0	12	1	2	0
2022-2023	1	0	0	0	1	0	0	0	0	0	0	0	0	0	0	0	0	0	0	0	8	0	4	0	0	3	1	0	1
Total	9	10	1	15	2	5	1	7	16	11	16	2	4	1	1	3	1	1	1	3	30	1	5	1	1	15	2	2	1

This supplementary material is hosted by Eurosurveillance as supporting information alongside the article "14 years of rotavirus A surveillance: unusual dominance of equine-like G3P[8] genotype with DS-1-like genotype constellation after the pandemic, Belgium, 2009 to 2023", on behalf of the authors, who remain responsible for the accuracy and appropriateness of the content. The same standards for ethics, copyright, attributions and permissions as for the article apply. Supplements are not edited by Eurosurveillance and the journal is not responsible for the maintenance of any links or email addresses provided therein.

Supplementary information S5

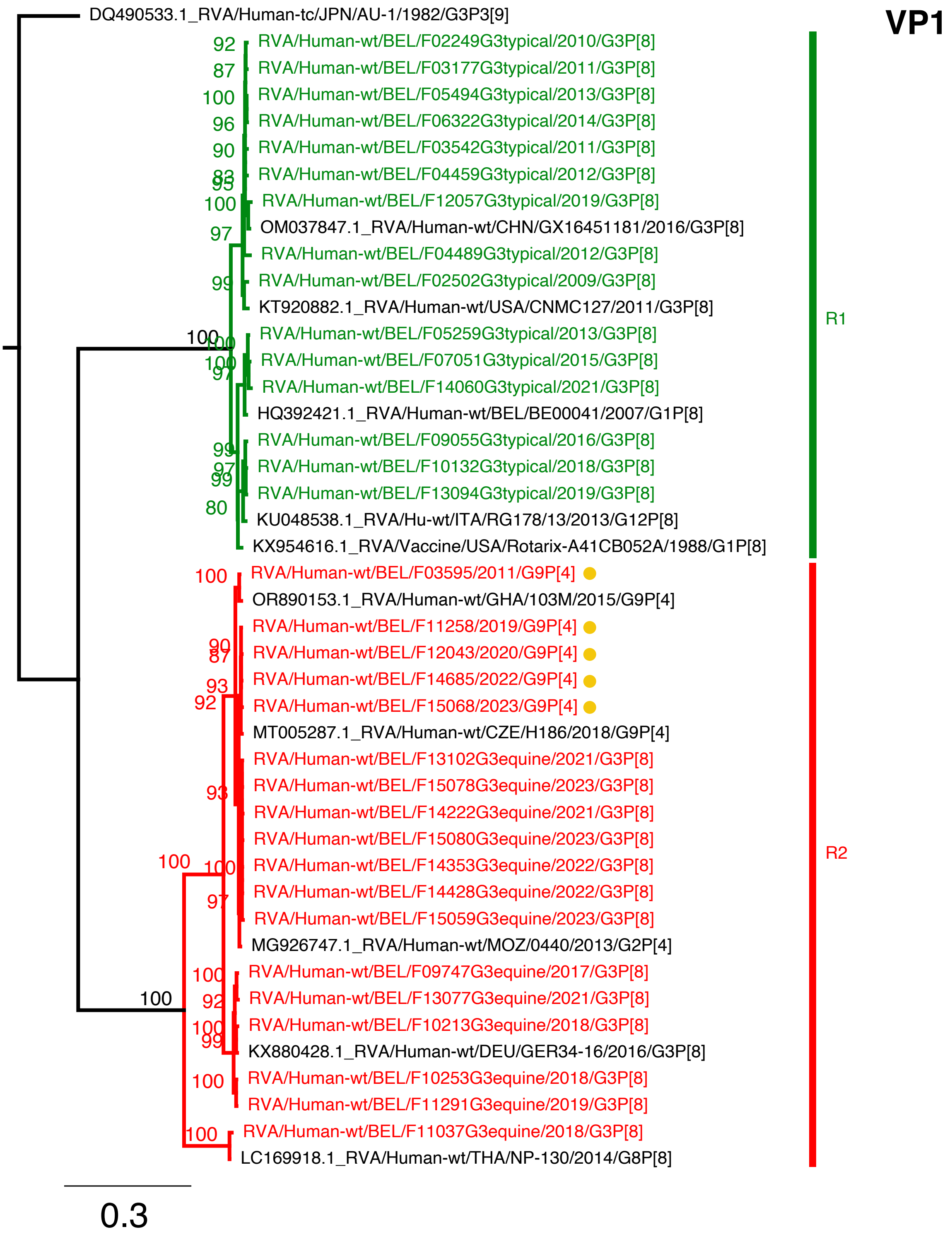

Supplementary information S5.1-11. Phylogenetic tree of coding sequence (nucleotide) of each segment, aligned with MAFFT v7 and ML tree constructed using IqTree 2.3.6. Red: DS-1-like segment. Green: Wa-like segment. Orange circle: G9P[4]. Black: Reference sequences from Genbank. **S5.1.** Phylogenetic tree of VP1.

This supplementary material is hosted by Eurosurveillance as supporting information alongside the article "14 years of rotavirus A surveillance: unusual dominance of equine-like G3P[8] genotype with DS-1-like genotype constellation after the pandemic, Belgium, 2009 to 2023", on behalf of the authors, who remain responsible for the accuracy and appropriateness of the content. The same standards for ethics, copyright, attributions and permissions as for the article apply. Supplements are not edited by Eurosurveillance and the journal is not responsible for the maintenance of any links or email addresses provided therein.

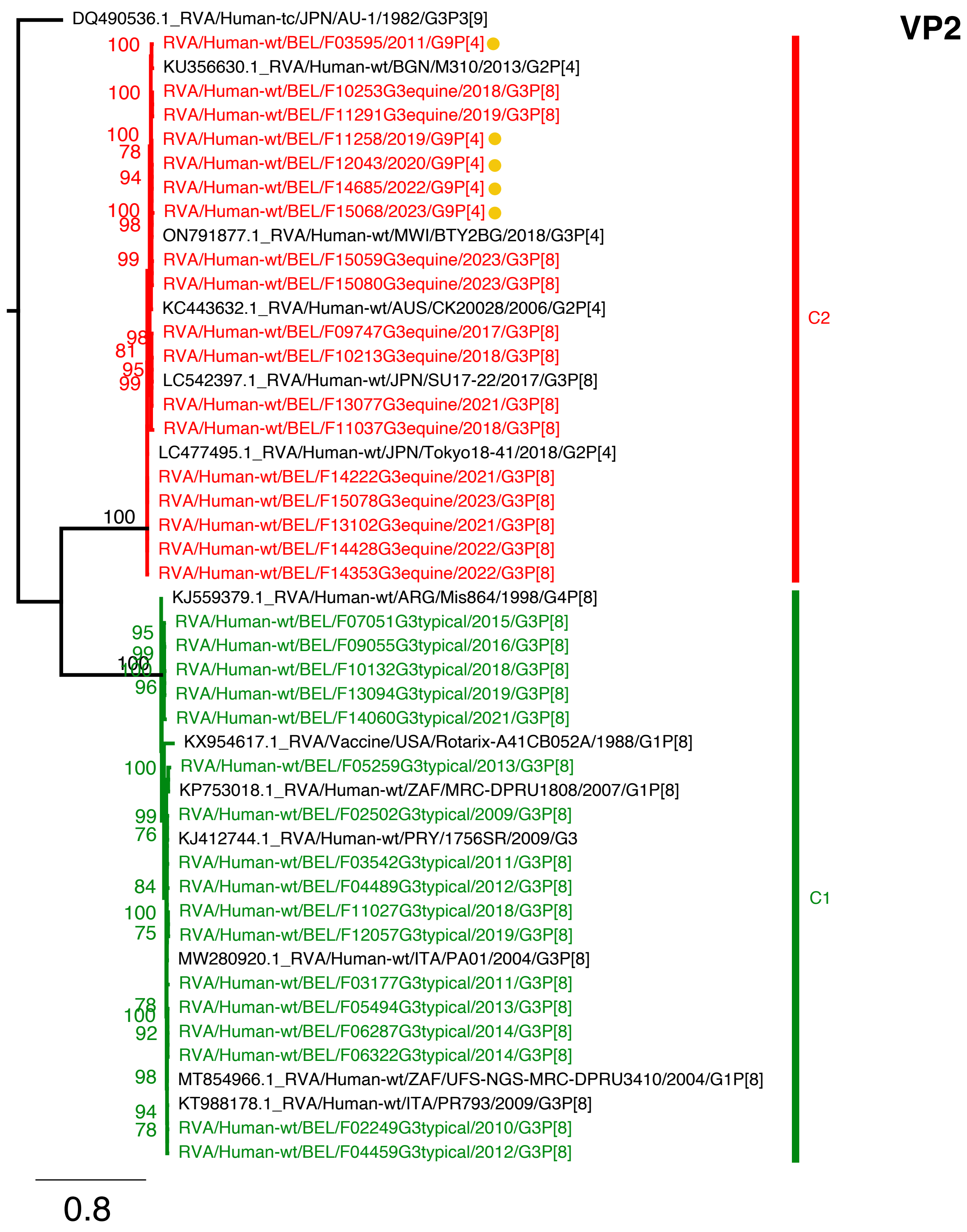

S5.2. Phylogenetic tree of VP2.

This supplementary material is hosted by Eurosurveillance as supporting information alongside the article "14 years of rotavirus A surveillance: unusual dominance of equine-like G3P[8] genotype with DS-1-like genotype constellation after the pandemic, Belgium, 2009 to 2023", on behalf of the authors, who remain responsible for the accuracy and appropriateness of the content. The same standards for ethics, copyright, attributions and permissions as for the article apply. Supplements are not edited by Eurosurveillance and the journal is not responsible for the maintenance of any links or email addresses provided therein.

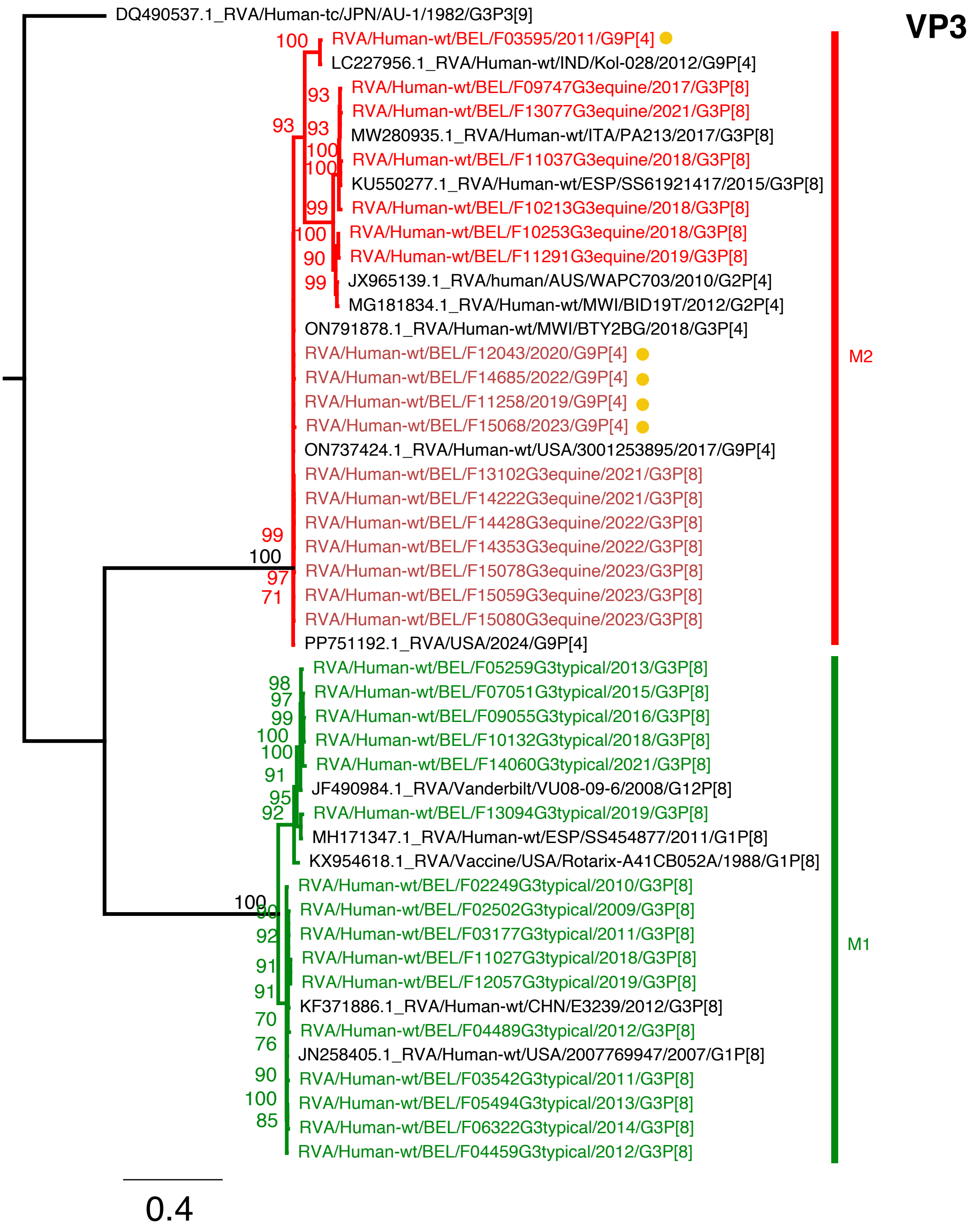

S5.3. Phylogenetic tree of VP3.

This supplementary material is hosted by Eurosurveillance as supporting information alongside the article "14 years of rotavirus A surveillance: unusual dominance of equine-like G3P[8] genotype with DS-1-like genotype constellation after the pandemic, Belgium, 2009 to 2023", on behalf of the authors, who remain responsible for the accuracy and appropriateness of the content. The same standards for ethics, copyright, attributions and permissions as for the article apply. Supplements are not edited by Eurosurveillance and the journal is not responsible for the maintenance of any links or email addresses provided therein.

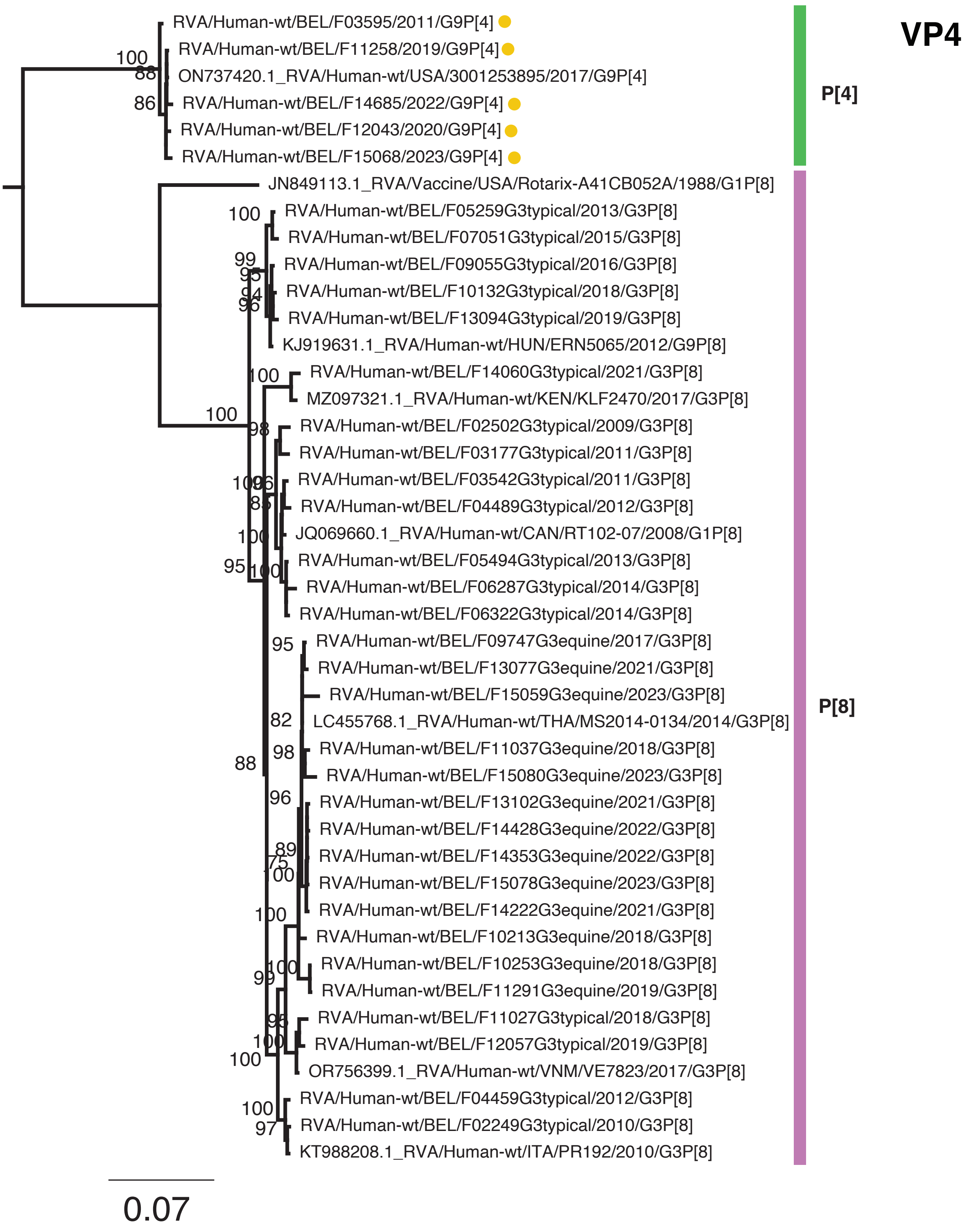

S5.4. Phylogenetic tree of VP4.

This supplementary material is hosted by Eurosurveillance as supporting information alongside the article "14 years of rotavirus A surveillance: unusual dominance of equine-like G3P[8] genotype with DS-1-like genotype constellation after the pandemic, Belgium, 2009 to 2023", on behalf of the authors, who remain responsible for the accuracy and appropriateness of the content. The same standards for ethics, copyright, attributions and permissions as for the article apply. Supplements are not edited by Eurosurveillance and the journal is not responsible for the maintenance of any links or email addresses provided therein.

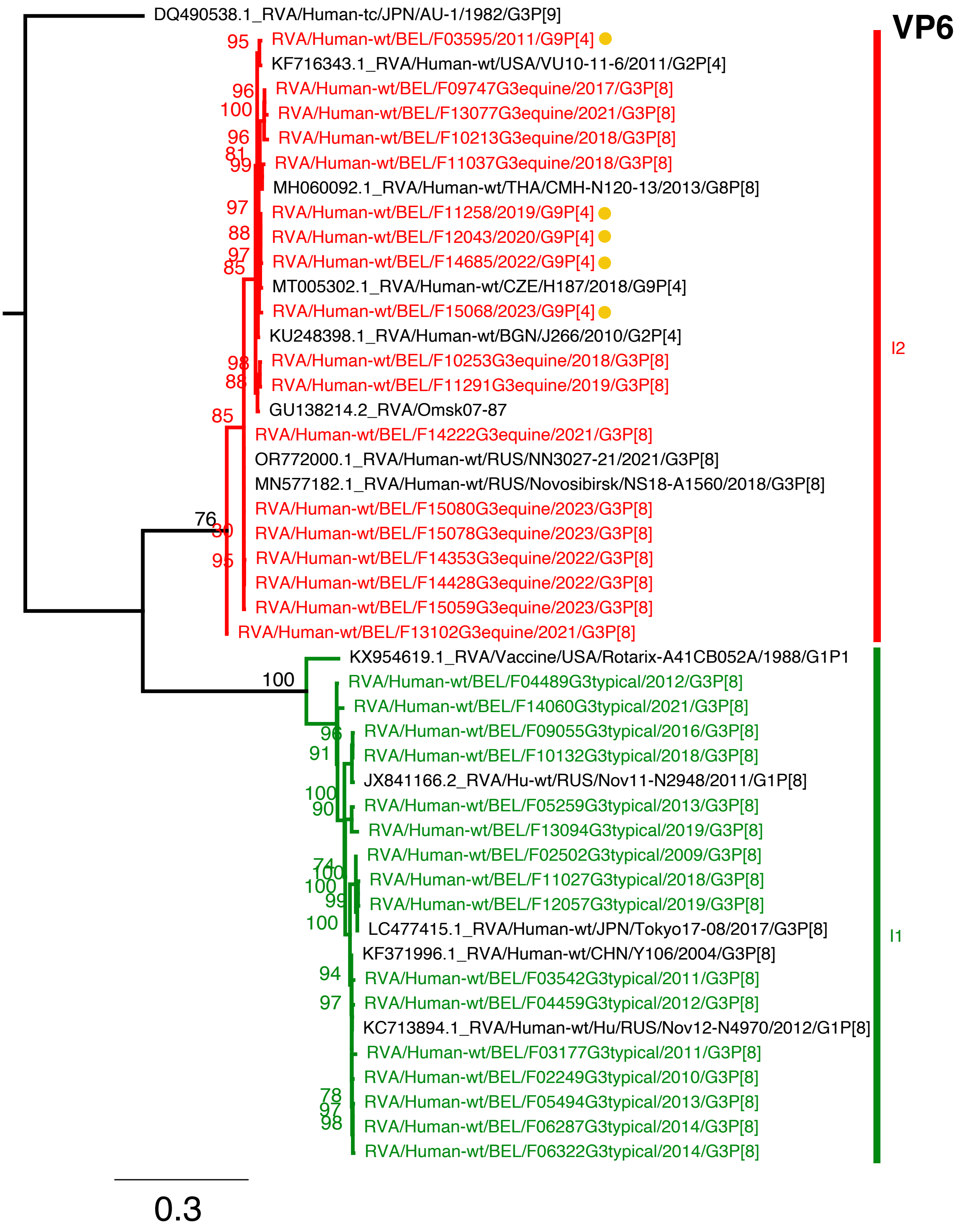

S5.5. Phylogenetic tree of VP6.

This supplementary material is hosted by Eurosurveillance as supporting information alongside the article "14 years of rotavirus A surveillance: unusual dominance of equine-like G3P[8] genotype with DS-1-like genotype constellation after the pandemic, Belgium, 2009 to 2023", on behalf of the authors, who remain responsible for the accuracy and appropriateness of the content. The same standards for ethics, copyright, attributions and permissions as for the article apply. Supplements are not edited by Eurosurveillance and the journal is not responsible for the maintenance of any links or email addresses provided therein.

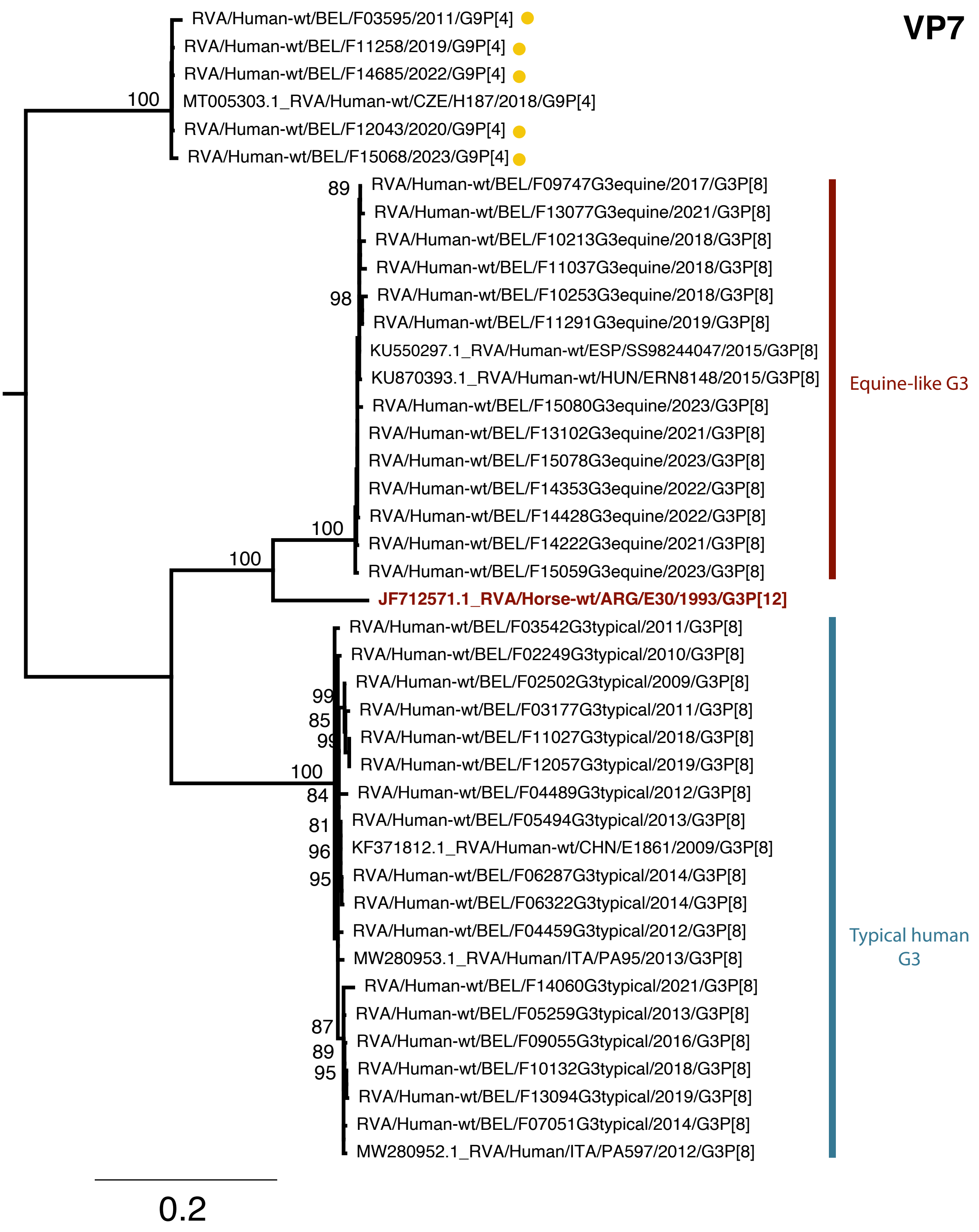

S5.6. Phylogenetic tree of VP7.

This supplementary material is hosted by Eurosurveillance as supporting information alongside the article "14 years of rotavirus A surveillance: unusual dominance of equine-like G3P[8] genotype with DS-1-like genotype constellation after the pandemic, Belgium, 2009 to 2023", on behalf of the authors, who remain responsible for the accuracy and appropriateness of the content. The same standards for ethics, copyright, attributions and permissions as for the article apply. Supplements are not edited by Eurosurveillance and the journal is not responsible for the maintenance of any links or email addresses provided therein.

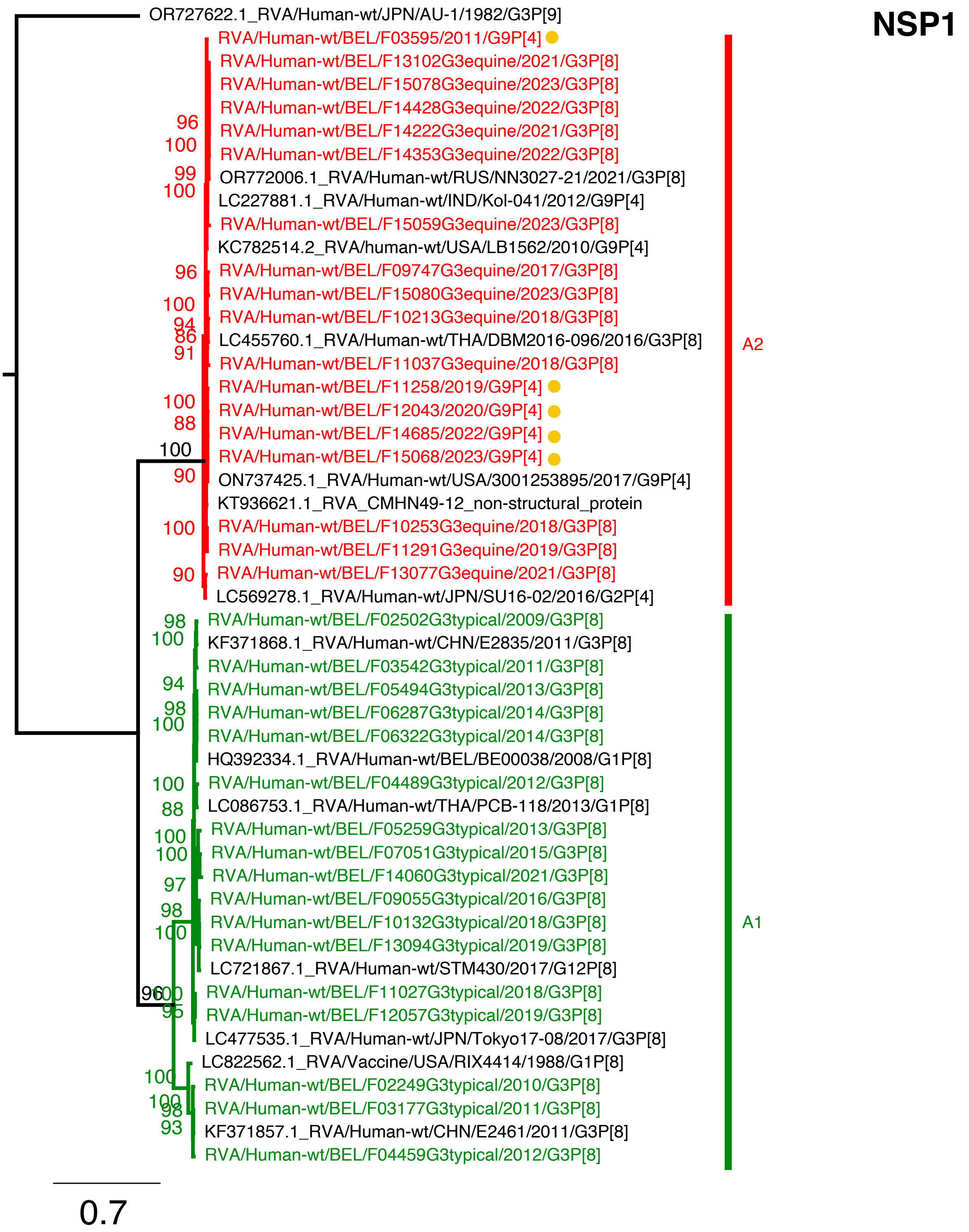

S5.7. Phylogenetic tree of NSP1.

This supplementary material is hosted by Eurosurveillance as supporting information alongside the article "14 years of rotavirus A surveillance: unusual dominance of equine-like G3P[8] genotype with DS-1-like genotype constellation after the pandemic, Belgium, 2009 to 2023", on behalf of the authors, who remain responsible for the accuracy and appropriateness of the content. The same standards for ethics, copyright, attributions and permissions as for the article apply. Supplements are not edited by Eurosurveillance and the journal is not responsible for the maintenance of any links or email addresses provided therein.

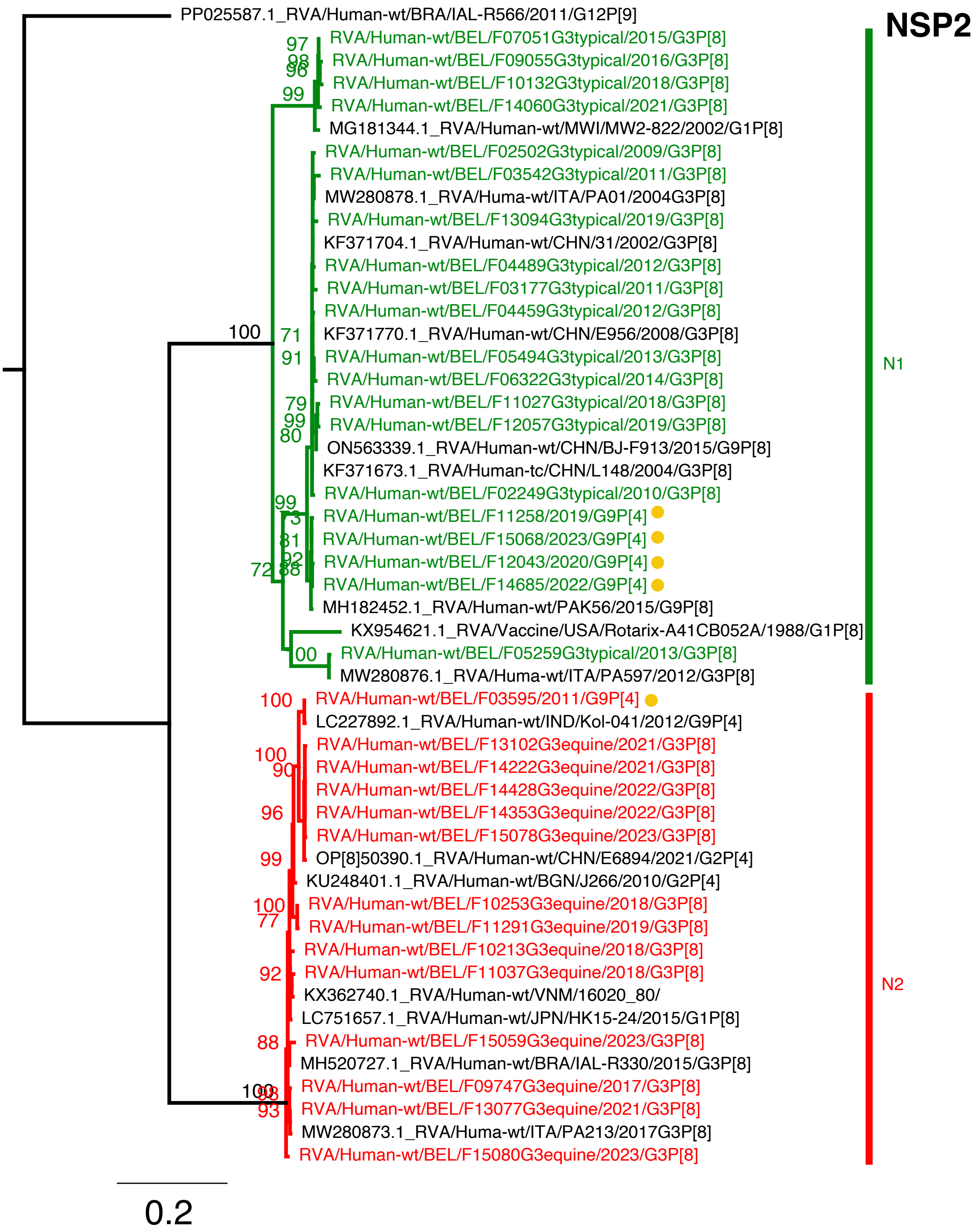

S5.8. Phylogenetic tree of NSP2.

This supplementary material is hosted by Eurosurveillance as supporting information alongside the article "14 years of rotavirus A surveillance: unusual dominance of equine-like G3P[8] genotype with DS-1-like genotype constellation after the pandemic, Belgium, 2009 to 2023", on behalf of the authors, who remain responsible for the accuracy and appropriateness of the content. The same standards for ethics, copyright, attributions and permissions as for the article apply. Supplements are not edited by Eurosurveillance and the journal is not responsible for the maintenance of any links or email addresses provided therein.

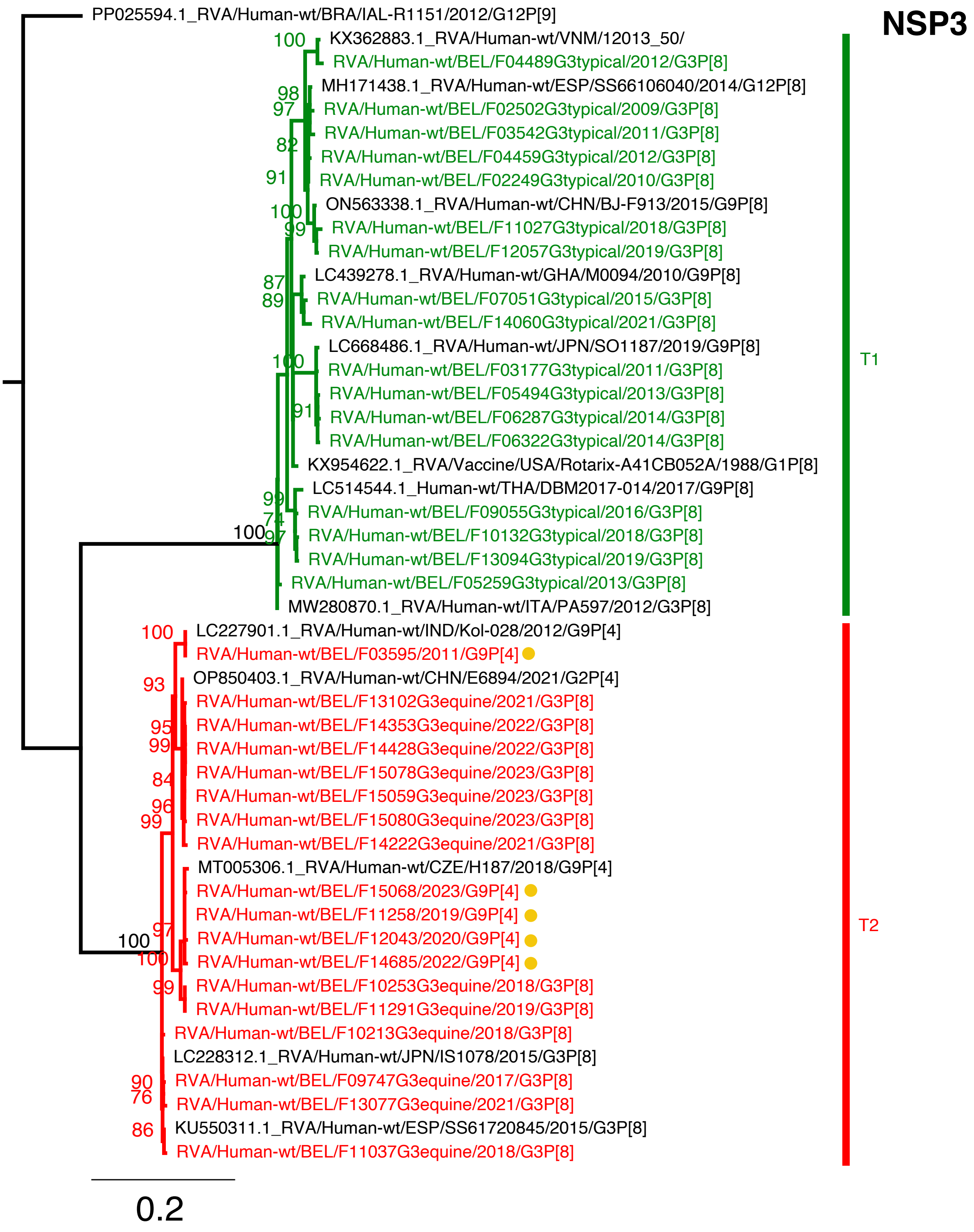

S5.9. Phylogenetic tree of NSP3.

This supplementary material is hosted by Eurosurveillance as supporting information alongside the article "14 years of rotavirus A surveillance: unusual dominance of equine-like G3P[8] genotype with DS-1-like genotype constellation after the pandemic, Belgium, 2009 to 2023", on behalf of the authors, who remain responsible for the accuracy and appropriateness of the content. The same standards for ethics, copyright, attributions and permissions as for the article apply. Supplements are not edited by Eurosurveillance and the journal is not responsible for the maintenance of any links or email addresses provided therein.

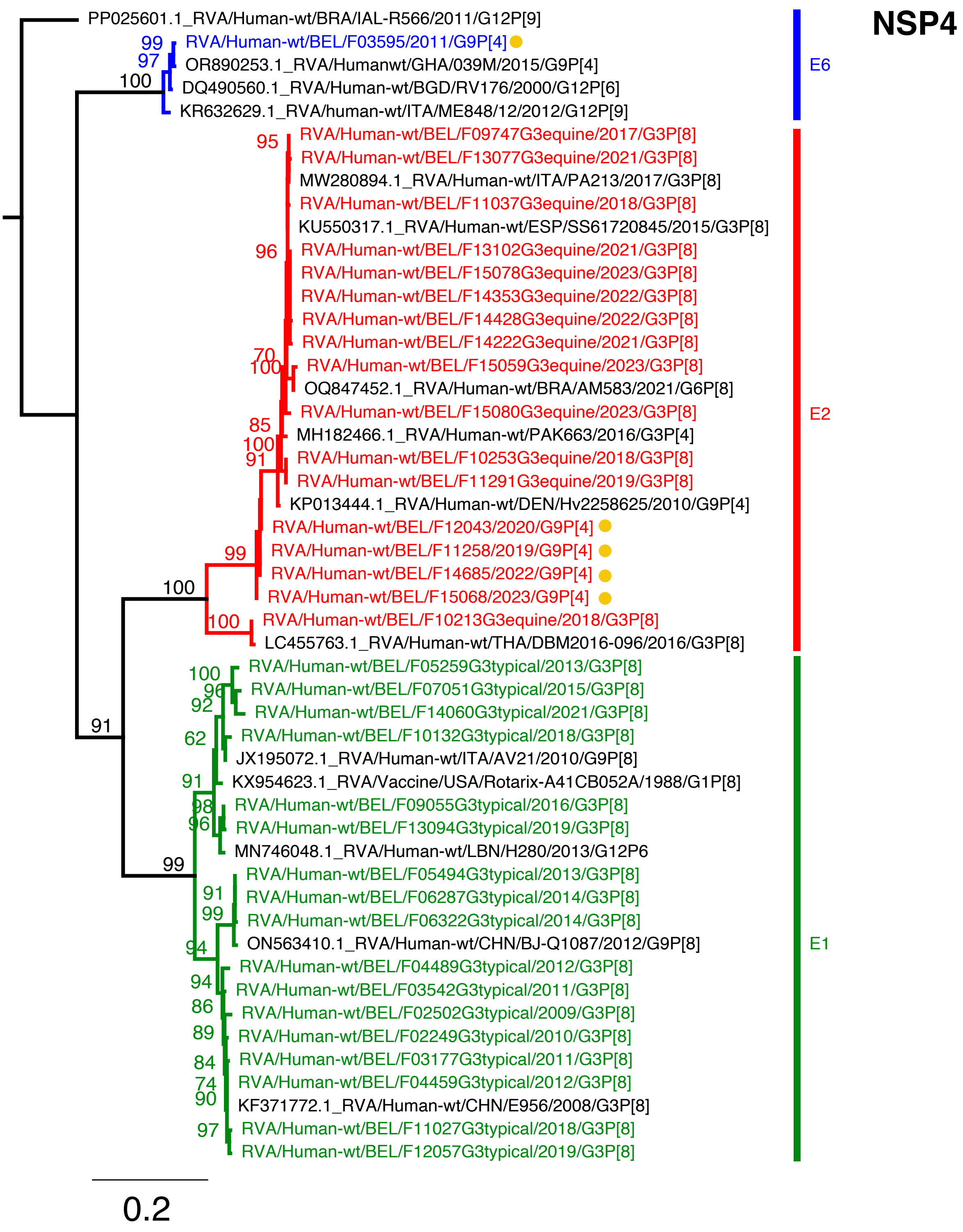

S5.10. Phylogenetic tree of NSP4.

This supplementary material is hosted by Eurosurveillance as supporting information alongside the article "14 years of rotavirus A surveillance: unusual dominance of equine-like G3P[8] genotype with DS-1-like genotype constellation after the pandemic, Belgium, 2009 to 2023", on behalf of the authors, who remain responsible for the accuracy and appropriateness of the content. The same standards for ethics, copyright, attributions and permissions as for the article apply. Supplements are not edited by Eurosurveillance and the journal is not responsible for the maintenance of any links or email addresses provided therein.

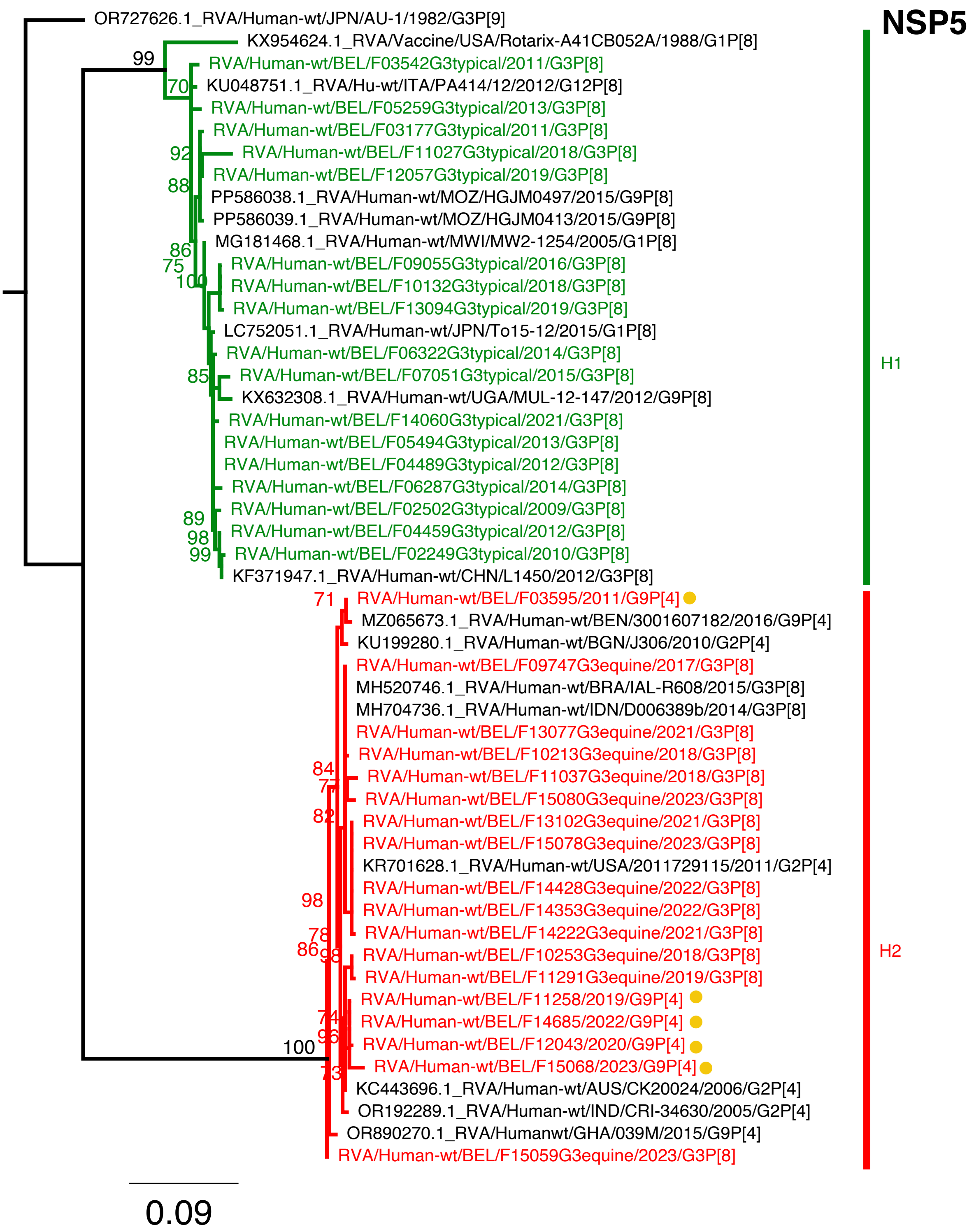

S5.11. Phylogenetic tree of NSP5.

This supplementary material is hosted by Eurosurveillance as supporting information alongside the article "14 years of rotavirus A surveillance: unusual dominance of equine-like G3P[8] genotype with DS-1-like genotype constellation after the pandemic, Belgium, 2009 to 2023", on behalf of the authors, who remain responsible for the accuracy and appropriateness of the content. The same standards for ethics, copyright, attributions and permissions as for the article apply. Supplements are not edited by Eurosurveillance and the journal is not responsible for the maintenance of any links or email addresses provided therein.

Supplementary information S6

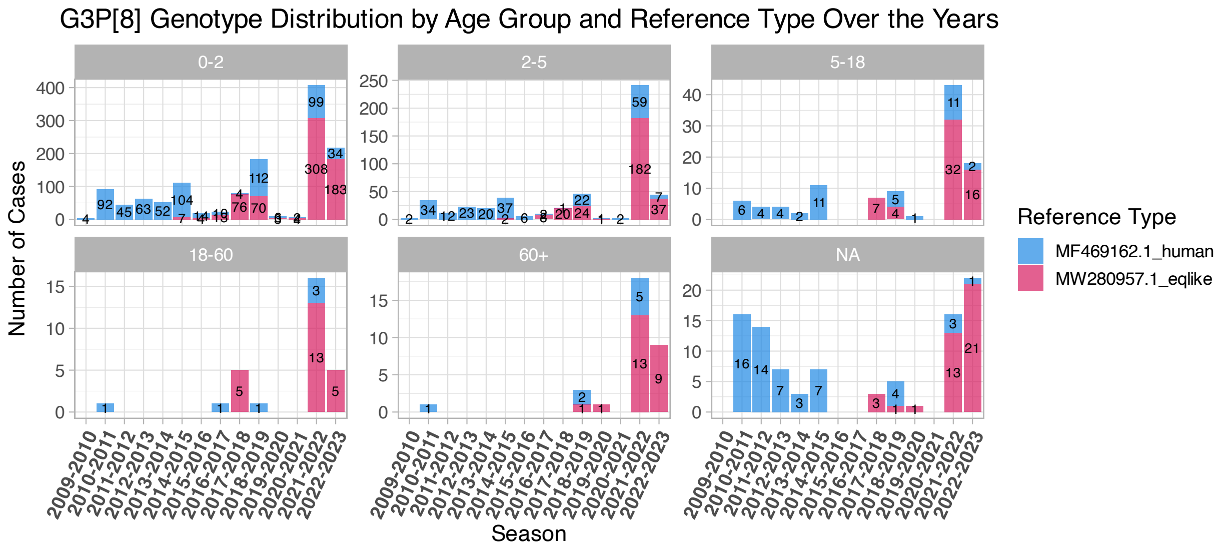

Supplementary information S6. Equine-like and typical human G3P[8] numbers over the years in different age groups.
